# Smoking-induced microbial dysbiosis: a key driver of systemic diseases and emerging therapeutic opportunities

**DOI:** 10.1038/s41420-025-02914-x

**Published:** 2025-12-10

**Authors:** Zihao Zhou, Xinyuan Zhao, Shuyu Sun, Li Cui

**Affiliations:** 1 Stomatological Hospital, School of Stomatology, Southern Medical University, Guangzhou, 510280 Guangdong China; 2 School of Dentistry, University of California, Los Angeles, Los Angeles, 90095 CA USA

**Keywords:** Microbiology, Diseases

## Abstract

Tobacco use, including both traditional and electronic cigarettes, profoundly alters host–microbiota interactions, contributing to the pathogenesis of various systemic diseases. Smoking-induced microbial dysbiosis impacts multiple anatomical sites, including the oral cavity, respiratory tract, and gastrointestinal system, driving disease progression through mechanisms such as immune modulation, chronic inflammation, and metabolic dysregulation. This review examines the disruption of microbial ecosystems by smoking, with a focus on the imbalance between beneficial and pathogenic microorganisms. In the oral cavity, smoking is strongly linked to diseases such as periodontitis and oral cancer, marked by shifts in microbial diversity and functional profiles. Similar dysbiotic changes are observed in the respiratory and gastrointestinal systems, where smoking impairs mucosal immunity, increases oxidative stress, and compromises barrier integrity, thereby enhancing susceptibility to chronic diseases. Additionally, the review addresses the challenges in establishing causality between microbial changes and disease outcomes, emphasizing the need for more comprehensive research utilizing multi-omics approaches and longitudinal studies. By exploring the potential for microbiota-based interventions, this review underscores the critical role of microbial dysbiosis in smoking-related health risks, providing valuable insights for the development of targeted therapeutic strategies to mitigate the global health burden of tobacco use.

## Facts

- **Causal link between microbial dysbiosis and disease outcomes**: The relationship between smoking-induced microbial imbalances (e.g., *Fusobacterium*, *Prevotella* enrichment) and disease progression (e.g., periodontitis, COPD, colorectal cancer) remains correlative. Mechanistic studies are needed to determine whether dysbiosis directly drives pathology or is a secondary effect of inflammation or epithelial damage.
- **Electronic vs. traditional cigarettes**: Comparative impacts of electronic cigarettes (ECs) and combustible cigarettes on microbial communities—such as EC-specific enrichment of *Veillonella* and suppression of *Porphyromonas*—warrant further investigation to clarify distinct disease risks (e.g., oral preneoplasia, periodontal inflammation).
- **Reversibility post-cessation**: Evidence suggests partial restoration of oral and gut microbiota after smoking cessation (e.g., *Neisseria* recovery in saliva, *Lactobacillaceae* increase with modified-risk products), but long-term ecological resilience and its health implications require longitudinal human cohort studies.
- **Gut–lung axis modulation**: Smoking-induced disruptions in short-chain fatty acid (SCFA) production and bile acid metabolism (e.g., taurodeoxycholic acid elevation) may link gut dysbiosis to pulmonary inflammation and cancer, emphasizing the need for multi-omics integration to decode systemic microbial cross-talk.

## Introduction

Globally, over 1 billion individuals smoke cigarettes, contributing significantly to the global health burden [1]. Chronic smoking is strongly associated with increased morbidity and a marked reduction in life expectancy, with an estimated loss of up to a decade of life in developing countries [2, 3]. Cigarette smoking (CS) is a well-established risk factor for a wide range of systemic diseases, including cardiovascular disorders, respiratory diseases, diabetes, inflammatory bowel diseases, and various cancers [4, 5]. Smoking exerts direct toxicological effects on the cardiovascular system, contributing to conditions such as atherosclerosis and coronary artery disease, and is a leading cause of chronic obstructive pulmonary disease (COPD) [6, 7]. Additionally, smoking is strongly linked to several cancers, including those of the oral cavity, upper digestive tract, lung, bladder, kidney, and liver. These associations are primarily driven by mechanisms such as the induction of somatic mutations, oxidative stress, and epigenetic modifications, which disrupt cellular function and promote disease progression. Importantly, smoking cessation can substantially reduce these health burdens, leading to significant improvements in overall health outcomes [8, 9].

**Fig. 1:**
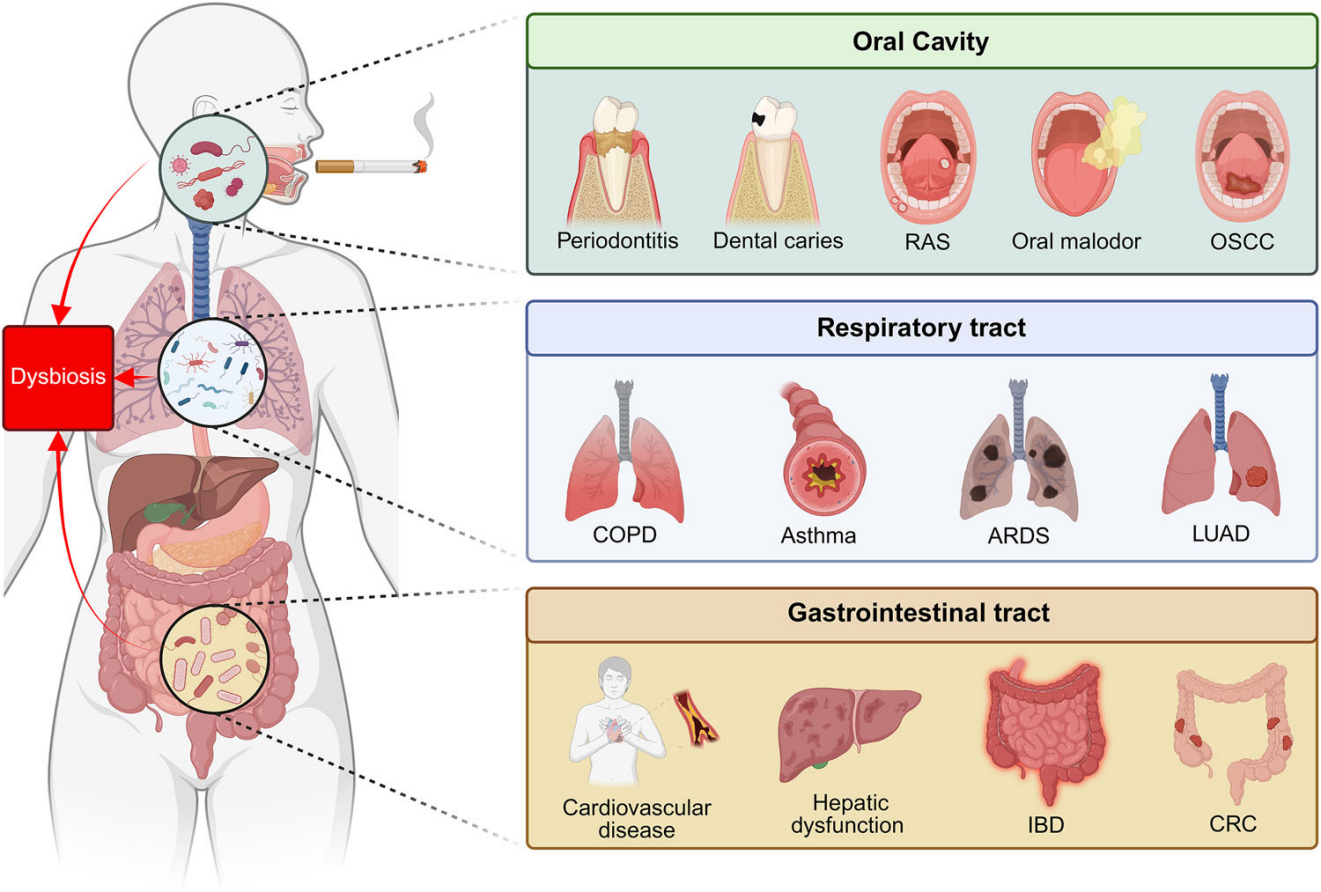
Smoking-induced microbial dysbiosis and its systemic impact across the oral, respiratory, and gastrointestinal tracts. Cigarette smoking disrupts the homeostasis of host-associated microbiota, leading to microbial dysbiosis that affects multiple organ systems. The oral, respiratory, and gastrointestinal tracts—each directly or indirectly exposed to tobacco smoke—exhibit distinct yet interconnected microbial alterations. In the oral cavity, dysbiosis contributes to periodontitis, dental caries, recurrent aphthous stomatitis (RAS), oral malodor, and oral squamous cell carcinoma (OSCC). In the respiratory tract, microbial imbalance exacerbates chronic obstructive pulmonary disease (COPD), asthma, acute respiratory distress syndrome (ARDS), and lung adenocarcinoma (LUAD). In the gastrointestinal tract, smoking-induced dysbiosis compromises mucosal barrier integrity, disrupts immune and metabolic homeostasis, and promotes cardiovascular disease, hepatic dysfunction, inflammatory bowel disease (IBD), and colorectal cancer (CRC). The red arrows indicate the bidirectional crosstalk between microbial dysbiosis and systemic disorders, highlighting how tobacco-induced microbial perturbations propagate through mucosal and immune pathways to influence distal organs. Created in https://BioRender.com.

Beyond its direct systemic toxicological effects, smoking exerts a profound impact on host-associated microbial communities across multiple body sites (Fig. 1). The human microbiota, composed of bacteria, fungi, archaea, and other microorganisms, plays a critical role in regulating immune responses, metabolism, and defense against harmful pathogens. Disruptions to this microbial ecosystem—termed dysbiosis—are implicated in a wide array of diseases [10–12]. Smoking has been shown to induce shifts in the microbiota across several body niches, including the oral cavity, respiratory tract, and gastrointestinal system. These microbial alterations contribute to the onset and progression of systemic diseases through various mechanisms, including immune modulation, chronic inflammation, metabolic dysregulation, oxidative stress, epigenetic modifications, and impaired barrier function.

In the oral cavity, smoking-induced dysbiosis plays a significant role in the development of oral diseases, such as periodontitis, by disrupting the balance of oral microbiota and promoting pathogenic species that drive inflammation and tissue destruction [13, 14]. In the respiratory tract, smoking exacerbates inflammation, leading to increased mucus production, impaired ciliary function, and airway remodeling, which worsen the pathophysiology of respiratory diseases [15, 16]. Within the gastrointestinal system, smoking alters several physiological functions, including the production of mucins, which are critical for mucosal protection, as well as the integrity of tight junctions in the small intestine, which compromises gut barrier function. This disruption can facilitate the translocation of harmful pathogens and toxins, further exacerbating inflammation [17, 18]. Moreover, smoking increases oxidative stress by inducing the production of reactive oxygen species (ROS), which directly damage cellular structures such as lipids, proteins, and DNA, contributing to tissue inflammation, cellular dysfunction, and the progression of chronic diseases. These oxidative effects are compounded by the immune dysregulation caused by microbial alterations [19, 20]. Collectively, these microbial, physiological, and oxidative changes triggered by smoking may amplify its detrimental impact on health, exacerbating a wide range of diseases and impairing the body’s ability to repair and protect itself.

Recent advances in metagenomic sequencing and other omics technologies have provided valuable insights into the specific microbial alterations associated with smoking-related diseases. These technologies have enabled the identification of distinct microbial signatures linked to various smoking-induced pathologies [21–23]. However, a fundamental challenge remains in determining whether these microbial changes are causally involved in disease development or if they represent secondary consequences of the pathological processes. Although strong correlations between smoking, pathophysiological outcomes, and microbial dysbiosis have been observed in both human and animal models, these associations alone do not establish causality. Further studies are essential to elucidate the mechanisms underlying these microbial shifts and to determine their role in disease onset and progression. Clarifying these relationships is critical for identifying potential therapeutic targets and developing strategies to mitigate the health impacts of smoking.

This review aims to provide a comprehensive overview of the impact of smoking on the microbiota across the oral, respiratory, and gastrointestinal tracts, and examines the crucial role these microbial alterations play in the pathogenesis of smoking-related diseases. By exploring the systemic effects of smoking on microbial communities, we discuss how these shifts contribute to the onset and exacerbation of a broad range of diseases, including cancer, cardiovascular conditions, and metabolic disorders. Furthermore, we highlight the mechanisms linking smoking-induced microbial dysbiosis to disease, emphasizing how disruptions in the microbiota of key organ systems can significantly influence disease pathophysiology. We also address the limitations of current research, particularly the challenge of distinguishing causality from correlation, and propose future research directions aimed at elucidating the mechanistic pathways involved. By investigating the microbiome’s role in smoking-related health risks, this review offers a novel perspective on potential therapeutic interventions, identifying promising avenues for future research that could lead to more effective strategies for mitigating the harmful effects of smoking on human health.

## Literature search methodology

This article is designed as a narrative review aiming to integrate current evidence on smoking-induced microbial dysbiosis and its systemic implications. To ensure a comprehensive and evidence-based synthesis, a literature search was conducted across PubMed, Scopus, and Web of Science databases. Publications from January 2010 to May 2025 were retrieved, with a particular emphasis on studies published within the past 5 years to capture the most recent advances. The search employed combinations of the following keywords: “cigarette”, “microbiome”, “dysbiosis”, “cancer”, “systemic diseases”, “COPD”, “periodontal disease”, and “diabetes”. Only peer-reviewed articles published in English were included. Eligible studies investigated the effects of tobacco exposure on microbial composition and its association with local or systemic pathophysiological outcomes, including inflammatory, metabolic, and neoplastic processes. Reviews, commentaries, conference abstracts, and studies not directly addressing tobacco-related microbial alterations were excluded. Additionally, reference lists of relevant publications were manually screened to identify supplementary studies. This comprehensive strategy aimed to provide an up-to-date and balanced synthesis of current evidence while minimizing potential selection bias.

## Overview of human microbiota

To understand how smoking disrupts host–microbe interactions, it is first necessary to outline the composition and function of the human microbiota. Cigarette smoke exerts distinct effects across the oral, respiratory, and gastrointestinal systems, each of which harbors unique microbial ecosystems essential for maintaining physiological homeostasis. A clear overview of these communities provides the foundation for interpreting how smoking-induced perturbations translate into local and systemic disease processes.

The human microbiota constitutes a vast and intricate assembly of microorganisms that inhabit various regions of the human body, playing an indispensable role in maintaining overall health and supporting critical physiological functions. This diverse community, which includes bacteria, viruses, fungi, and archaea, has co-evolved with humans over millennia, forming complex ecosystems that are essential for processes such as nutrient absorption, immune modulation, and pathogen defense [24–26]. Microorganisms within the microbiota assist in the digestion of food, the synthesis of essential vitamins, and the fermentation of fibers into short-chain fatty acids, which are important energy sources for the host. Furthermore, they contribute to the development and regulation of the immune system, promoting immune tolerance and defending against pathogenic microbes. The composition and balance of the microbiota are influenced by a variety of factors, including host genetics, diet, environmental exposures, and lifestyle choices [27, 28].

Distinct microbiomes have developed across various anatomical regions of the human body, each exhibiting unique characteristics and functions that collectively support overall health. The oral microbiome, first observed by Antonie van Leeuwenhoek in 1670 using his self-designed microscope, is the second-most diverse microbiome after the gut microbiome. Over 700 bacterial species have been identified within the oral cavity, which is subdivided into numerous niches, including saliva, the hard and soft palates, the tongue, lips, cheeks, and dental plaque biofilms [29, 30]. These distinct regions harbor specific microbial communities, which play vital roles in maintaining oral health, such as aiding in the digestion of food and protecting against pathogenic invaders. Disruption of the oral microbiome’s balance, a condition known as oral dysbiosis, is strongly associated with a range of oral diseases, including periodontitis and dental caries. The shift towards a pathogenic microbial profile often exacerbates inflammation, leading to tissue destruction and further compromising oral health [31, 32]. Recent research has highlighted the emerging links between oral dysbiosis and systemic diseases. The mechanisms behind these associations suggest that oral bacteria and their metabolites can enter the bloodstream through oral epithelial barriers or via direct interactions with the respiratory and digestive systems, thereby influencing systemic health [33–35]. Thus, the oral microbiome serves not only as a critical determinant of oral health but also as a potential modulator of broader health outcomes.

Historically, the respiratory tract was considered a sterile environment, with no microbial life present, a belief that endured for centuries. However, the advent of modern sequencing technologies, particularly 16S rRNA analysis, has revolutionized our understanding of the respiratory system. These advancements have demonstrated that the respiratory tract is not devoid of microorganisms, but rather supports a complex and diverse microbiome across both the upper and lower airways [36–38]. These respiratory microbiomes are shaped by a variety of factors, including environmental exposures, host genetics, and overall health status. Recent insights into the respiratory microbiota highlight its essential role in mucosal immunity and the regulation of inflammatory responses, which are key to maintaining respiratory health. Disruptions or imbalances in this microbiome—referred to as respiratory dysbiosis—have been linked to a range of chronic respiratory conditions, including asthma, COPD, and recurrent respiratory infections [39–41]. This growing body of evidence underscores the importance of the respiratory microbiota in both disease pathogenesis and the maintenance of pulmonary homeostasis, revealing its potential as a target for therapeutic interventions in respiratory diseases.

The gut microbiome is one of the most complex and densely populated microbial ecosystems in the human body, with trillions of microorganisms colonizing both the large and small intestines. This intricate community plays a central role in nutrient absorption, as well as the synthesis of essential metabolites such as short-chain fatty acids (SCFAs), which are crucial for maintaining intestinal health and regulating gut motility [42–44]. In addition to its metabolic functions, the gut microbiota exerts a profound influence on both local and systemic immune responses, shaping immune system development and regulating inflammatory pathways. Alterations in the composition of the gut microbiome, a condition known as gut dysbiosis, have been strongly associated with a range of diseases, including inflammatory bowel disease (IBD), metabolic syndrome, and various cancers. Disruptions in microbial diversity or the overgrowth of pathogenic species can compromise gut barrier function, facilitate systemic inflammation, and contribute to disease progression [45–47]. These findings underscore the pivotal role of the gut microbiome not only in intestinal health but also in the pathogenesis of a broad spectrum of systemic diseases, highlighting its potential as both a therapeutic target and a biomarker for disease detection.

Collectively, these interrelated microbial communities are essential for both localized functions and systemic homeostasis. Lifestyle factors, particularly smoking, have emerged as significant disruptors of these ecosystems. CS alters the composition and diversity of the microbiota across multiple body sites, impairing microbial balance and contributing to disease pathogenesis. Understanding the specific mechanisms by which smoking induces microbial dysbiosis across different anatomical sites is crucial, as this knowledge may uncover novel pathways for disease prevention, early detection, and personalized treatment. Moreover, such insights could inform the development of targeted therapeutic strategies aimed at restoring microbial balance, offering promising opportunities for improving health outcomes in individuals affected by smoking-related diseases.

## Interplay between cigarette smoking and the oral microbiome

Building upon the foundational understanding of human microbial ecosystems, the oral cavity represents the first and most directly exposed site to cigarette smoke, making it a critical interface for host–microbe–toxin interactions. The unique ecological complexity of the oral microbiome renders it highly sensitive to chemical and thermal insults from tobacco products. CS and its alternatives—including electronic and smokeless forms—profoundly reshape microbial composition, functional capacity, and host immune responses. These alterations not only initiate local inflammatory and metabolic disturbances but also propagate systemic effects through microbial translocation and immune modulation. The following sections delineate how different tobacco products perturb the oral microbiota, contributing to disease onset, progression, and potential reversibility upon cessation.

### Traditional cigarettes

**Fig. 2:**
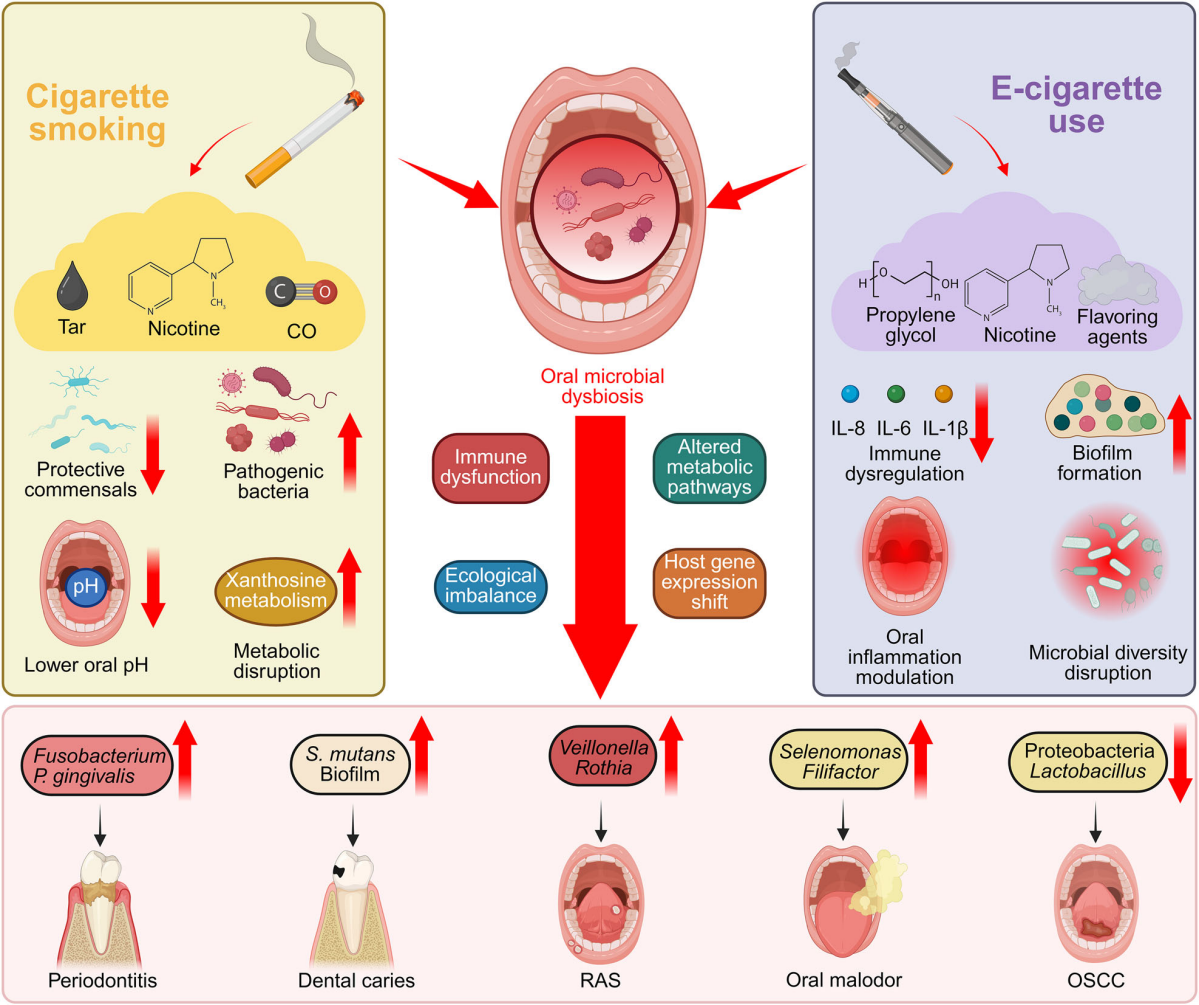
Distinct but converging impacts of cigarette smoking and e-cigarette use on oral microbial dysbiosis and disease development. Both conventional and electronic cigarettes induce oral microbial dysbiosis through overlapping yet distinct mechanisms. Cigarette smoke, composed mainly of tar, nicotine, and carbon monoxide (CO), perturbs oral microbial homeostasis by reducing protective commensal bacteria, enriching pathogenic species, lowering oral pH, and disrupting metabolic processes such as xanthosine metabolism. In contrast, e-cigarette aerosols containing propylene glycol, nicotine, and flavoring agents promote microbial diversity shifts, enhance biofilm formation, and trigger immune dysregulation characterized by elevated pro-inflammatory cytokines (IL-6, IL-8, and IL-1β). These disturbances converge on oral microbial dysbiosis marked by ecological imbalance, metabolic disruption, immune dysfunction, and altered host gene expression. Collectively, these changes facilitate the onset and progression of oral diseases, including periodontitis, dental caries, recurrent aphthous stomatitis (RAS), oral malodor, and oral squamous cell carcinoma (OSCC). Created in https://BioRender.com.

Traditional cigarettes profoundly influence the oral microbiome (Fig. 2 and Table 1). CS reduced microbial α-diversity and reshaped bacterial composition. Smokers showed increased abundance of *Moryella*, *Bulleidia*, *Moraxella*, and nitrite-producing species such as *Actinomyces* and *Veillonella*, which enhanced oral acidity. Smoking also upregulated pathways related to amino acid and nucleotide sugar metabolism. Co-occurrence analysis revealed positive correlations among smoker-enriched taxa and negative correlations with depleted ones, indicating that CS disrupted microbial networks and metabolic balance [48]. Heavy smoking further modified the oral microbiota, enriching *Veillonella dispar*, *Leptotrichia spp*., and *Prevotella pleuritidis*, while nicotine dependence was linked to higher levels of *Streptobacillus hongkongensis*, *Fusobacterium massiliense*, and *Prevotella bivia*. Functional profiling showed increased activity in tricarballylate utilization, lactate racemization, and xanthosine metabolism pathways [49]. Shotgun metagenomic data confirmed that smoking induces salivary dysbiosis, with elevated *Prevotella* and *Megasphaera* and reduced *Neisseria*, *Oribacterium*, *Capnocytophaga*, and *Porphyromonas*. The enrichment of *Prevotella*, often linked to inflammation and oral cancer, underscored the pathogenic potential of smoking-altered communities [50]. CS also increased *Streptococcus mutans*, *Veillonella tobetsuensis*, and *Veillonella dispar* while decreasing *Lactobacillus* species. Notably, *S. mutans* strains from smokers displayed enhanced anthracene biodegradation, whereas *Lactobacillus* strains showed variable activity. Co-culture experiments revealed that bacterial interactions suppressed anthracene degradation, suggesting that smoking-induced dysbiosis altered both microbial composition and metabolic function [51]. Smoking further reduced oropharyngeal diversity and enriched periodontal pathogens such as *Bacillus* and *Burkholderia*. Unlike the nasopharyngeal microbiota, which remained relatively stable, the oropharyngeal microbiome exhibited marked smoking-related alterations, reflecting its higher sensitivity to CS exposure [52].

**Table 1:** Tobacco product-induced oral microbiota dysbiosis and disease implications.

Exposure category	Increased microbiota	Decreased microbiota	Functional/pathogenic alterations	Potential health implications	Ref
**Traditional cigarette**	*Moryella*, *Bulleidia*, *Moraxella*, *Actinomyces*, *Veillonella*	-	Increased oral acidity, altered amino acid metabolism and nucleotide sugar processing	Oral dysbiosis	[48]
**Traditional cigarette**	*V. dispar*, *Leptotrichia spp*., *P. pleuritidis*, *S. hongkongensis*, *F. massiliense*, *P. bivia*	-	Tricarballylate utilization, lactate racemization, and xanthosine metabolism ↑	Respiratory illness, cancer predisposition	[49]
**Traditional cigarette**	*Prevotella*, *Megasphaera*	*Neisseria*, *Oribacterium*, *Capnocytophaga*, *Porphyromonas*	Pro-inflammatory shifts, microbial dysbiosis, immune imbalance	Inflammatory diseases, oral cancer	[50]
**Traditional cigarette**	*S. mutans*, *V. tobetsuensis*, *V. dispar*	*Lactobacillus spp*.	Altered biodegradation activity of anthracene, microbial functional shifts, microbe–microbe interaction disruption	Oral dysbiosis, potential carcinogen retention	[51]
**Traditional cigarette**	*Bacillus*, *Burkholderia*	Oropharyngeal diversity	Impaired mucosal defense, disrupt microbial balance	Periodontal and respiratory inflammation	[52]
**Traditional cigarette**	*Fusobacterium*, *Campylobacter*	*Actinobacteria, Leptotrichia*, *Actinomyces*, *Corynebacterium*, *Lautropia*	Altered innate immune responses, reduced abundance of beneficial taxa	Periodontitis	[53]
**Traditional cigarette**	*F. nucleatum*, *N. sicca*, *V. dispar*	*Prevotella spp*.	Impacted subgingival bacterial ecology, reduced abundance of beneficial taxa	Periodontitis	[54]
**Traditional cigarette**	*A. odontolyticus*	*S. sanguinis*	Altered subgingival microbiota, increased disease susceptibility	Periodontal disease progression	[55]
**Traditional cigarette**	*Treponema*, *Selenomonas*, *Dialister*, *Atopobium*, *Veillonella*, *Filifactor*	*Cardiobacterium, Granulicatella*	Smoking dose-dependent microbial dysbiosis, increased abundance of periodontitis-associated taxa	Periodontitis, halitosis	[56]
**Traditional cigarette and Medwakh**	*S. spp*. (low caries), *Lactobacillus spp*. (high caries), *K. pneumoniae* (high caries)	-	Early cariogenic colonization, altered supragingival microbiome composition	Dental caries	[57]
**Traditional cigarette**	*Veillonella*, *Rothia*, *Sneathia*	*Bacteroidales*, *Bacteroides*	Promoted inflammatory environment, exacerbated mucosal ulceration	RAS	[58]
**Traditional cigarette**	*Staphylococcus*	Bacteroidetes, Proteobacteria, *Lactobacillus*	Microbiome–host gene interactions (CD74, PPP1R3C), weakened microbial homeostasis, enhanced risk of inflammation or infection	OSCC	[59]
**Electronic cigarette**	*S. pneumoniae*, *S. pyogenes*, *S. aureus*, *C. xerosis*	-	Disrupted microbial homeostasis, reduced resident plaque flora	Oral dysbiosis	[60]
**Electronic cigarette**	*Porphyromonas*, *Veillonella*	-	Elevated IL-6 and IL-1β, increased susceptibility to infection	Periodontitis	[61]
**Electronic cigarette**	*Abiotrophia, Aggregatibacter, Eikenella, Granulicatella*	-	Increased virulence and inflammatory response, altered biofilm architecture	Oral inflammation	[62]
**Electronic cigarette**	*Fusobacterium*, *Bacteroidales* (G-2), *Treponema*, *Porphyromonas*	-	Increased cytokine levels, promoted periodontal inflammation	Periodontitis	[63]
**Electronic cigarette**	*S. mutans*	*S. sanguinis*, *S. gordonii*	Enhanced biofilm formation, increased hydrophobicity and coaggregation	Caries, periodontitis	[64]
**Electronic cigarette**	*Filifactor*, *Treponema*, *Fusobacterium*, *P. gingivalis, F. nucleatum*	-	Elevated pro-inflammatory cytokines, cytokine correlations	Oral dysbiosis, Periodontal disease	[65]
**Electronic cigarette**	*Bacteroidetes_[G-3], Olsenella, Lachnospiraceae_[G-7], Filifactor*	*Bergeyella, Neisseria, Enterococcus and Haemophilus*	Altered 71 KEGG pathways, increased gingival inflammation	Periodontal disease	[66]
**Electronic cigarette**	*S. aureus*	-	Enhanced biofilm formation, suppressed immune response	Periodontitis, oral preneoplasia	[67]
**Electronic cigarette**	*Actinobacteria, Bacilli, Bacteroidia, Clostridia*	*Proteobacteria*	Increased cancer-related metabolites	Oral carcinogenesis, epithelial inflammation	[68]
**Electronic cigarette and nicotine pouch**	*P. gingivalis, T. forsythia, P. intermedia, F. nucleatum*	*-*	Increased salivary periodontopathogens	Periodontal disease	[69]
**Electronic cigarette and traditional cigarette**	*Veillonella*	*-*	Altered β-diversity	Periodontitis, oral dysbiosis	[70]
**Electronic cigarette and traditional cigarette**	*Actinomyces, Prevotella (CS)* *Veillonella (EC)*	*Porphyromonas, Peptostreptococcus (EC)*	Promoted anaerobic growth, oral flora imbalances	Dental caries, halitosis	[71]
**Smokeless tobacco**	*Pichia*, *Starmerella*, *Fusarium*	*Alternaria*	Fungal dysbiosis, increased pathotrophic activity	Oral carcinogenesis	[72]
**Smokeless tobacco**	*E. nodatum, P. micros, S. anginosus, S. constellatus*	*V. parvula*	Disrupted the oral microbial balance, protective flora suppression	Oral ecology disruption, periodontitis	[73]
**Smokeless tobacco**	*Firmicutes*, *Streptococcus*, *Actinomyces*	*Bacteroidetes*, *Fusobacteria*	Dose-dependent variation in oral microbial diversity	Periodontal disease, oral health deterioration	[74]
**Smokeless tobacco**	*Prevotella*, *Fusobacterium*, *Veillonella*	-	Functional shifts in nitrogen, nucleotide, energy metabolism	OPL	[75]
**Smokeless tobacco**	*Staphylococcus*, *Fusobacterium*, *Campylobacter*	-	Enriched nitrosamine-producing bacteria	OSCC	[76]
**Smokeless tobacco**	*Prevotella*, *Capnocytophaga*, *Fusobacterium*	-	Increased LPS biosynthesis pathway	OSCC, oral inflammation	[77]
**Smokeless tobacco**	*Staphylococcaceae*, *Corynebacterium_1*, *Cardiobacterium*	*Lactobacillus*, *Candida*, *Prevotella*	Altered oral microbiome spatiality	Oral carcinogenesis, OPL	[78]
**Smokeless tobacco**	*R. mucilaginosa*, *S. sp*. *oral taxon 66*, *A. meyeri*	*O. asaccharolyticum*	Increased acetaldehyde-production potential	Oral carcinogenesis	[79]
**Smokeless tobacco and traditional cigarette**	*Firmicutes, N. subflava and P. endodontalis*	Proteobacteria, *Actinomyces, Haemophilus, Neisseria, Rothia, Veillonella*	Altered α-diversity, deplete oxygen, increase salivary pH	Oral infections, inflammation	[80]
**Smokeless tobacco and traditional cigarette**	*F. spp., S. spp., Shuttleworthia (CS)* *Fusobacterium, Catonella, Fretibacterium*	*-*	Increased glutamate and aspartate biosynthesis	Oral carcinogenesis	[81]

Smoking-induced dysbiosis is a key driver of oral disease, particularly periodontitis. CS decreased *Actinobacteria* while increasing gram-negative anaerobes such as *Fusobacterium* and *Campylobacter*. Smokers showed lower abundance of *Leptotrichia*, *Actinomyces*, *Corynebacterium*, and *Lautropia*, indicating a microbial shift favoring periodontal pathogenesis [53]. Similarly, tobacco use altered the subgingival microbiome in chronic periodontitis, enriching *Fusobacterium nucleatum*, *Neisseria sicca*, and *Veillonella dispar* while depleting *Prevotella* species. These changes increase diversity yet promote dysbiosis, accelerating disease progression [54]. CS also elevated *Actinomyces odontolyticus* and reduced *Streptococcus sanguinis*, disrupting the balance between pathogenic and protective bacteria [55]. Moreover, CS altered microbial niches at multiple oral sites, enriching *Treponema* and *Selenomonas* in saliva and *Dialister* and *Atopobium* on the tongue. Genera such as *Veillonella* and *Filifactor* were found to correlate with cumulative exposure and were associated with periodontitis and halitosis, suggesting that the tongue might serve as a reservoir for pathogenic taxa in smokers [56].

Accumulating evidence indicates that smoking-related dysbiosis extends beyond periodontitis, contributing to caries, recurrent aphthous stomatitis (RAS), and oral squamous cell carcinoma (OSCC). Cigarette and alternative tobacco use markedly alter the supragingival microbiome: *Streptococcus* species dominated in smokers with low caries, while *Lactobacillus* species increased in those with high caries indices. Medwakh smokers showed enrichment of periodontopathogens in low-caries subjects and higher *Klebsiella pneumoniae* levels in high-caries subjects, highlighting microbiota-based differences in caries susceptibility [57]. Tobacco smoking also aggravated dysbiosis in RAS, reducing diversity and altering taxa composition. Smokers with RAS exhibited elevated *Veillonella*, *Rothia*, and *Sneathia* and reduced *Bacteroidales* and *Bacteroides*. These microbial changes correlated with smoking frequency and activated pathways related to respiration and pathogenicity, indicating that CS exacerbated RAS through microbial modulation [58]. During OSCC development, CS decreased microbial diversity and reduced Bacteroidetes, Proteobacteria, and *Lactobacillus* while promoting *Staphylococcus* proliferation. Integrative analyses showed associations between microbial composition and host gene expression, such as CD74 positively correlated with *Lactobacillus* and PPP1R3C negatively with Bacteroidota, suggesting that smoking-induced microbial shifted might interact with host transcriptional programs to promote OSCC progression [59].

### Electronic cigarettes

Electronic cigarettes (ECs), developed as a safer alternative to traditional cigarettes, were initially hypothesized to reduce nicotine dependence and diminish adverse health effects due to the absence of tar and incomplete combustion byproducts. However, recent findings have highlighted that EC aerosols, containing nicotine, propylene glycol, and flavoring agents, disrupt the oral microbiome equilibrium while also promoting oral inflammatory responses, potentially exacerbating oral tissue damage. For instance, the use of electronic smoking devices altered dental microbiocenosis, reducing resident plaque microflora and increasing colonization by opportunistic pathogens such as *Streptococcus pneumoniae* and *S. pyogenes*. Heating tobacco systems and vape use shifted microbial profiles, with higher frequencies of *S. aureus* and *Corynebacterium xerosis*, potentially impacting oral health through transient pathogen colonization [60]. In addition, EC use altered oral microbiota, increasing *Porphyromonas* and *Veillonella* abundance and elevating IL-6 and IL-1β levels, suggesting heightened inflammatory responses. Exposure to EC aerosols enhanced susceptibility to infection, as shown by increased inflammatory responses in *Porphyromonas gingivalis-* and *Fusobacterium nucleatum-*challenged epithelial and malignant cell lines, highlighting a dysbiotic microbiome and immune dysregulation in EC users [61]. Remarkably, EC use led to pathogen overrepresentation, increased virulence, and a strong pro-inflammatory response in the oral cavity, comparable to severe periodontitis. The carbon-rich glycol/glycerol vehicle in ECs altered biofilm architecture within 24 h of exposure. A machine-learning classifier based on metagenomic signatures effectively identified EC users, including those who use ECs alone or alongside cigarettes, raising concerns about the safety of ECs and challenging harm reduction claims [62]. Similarly, the use of EC altered the subgingival microbiome, enriching *Fusobacterium* and *Bacteroidales* (G-2), and shared similarities with the microbiomes of conventional smokers and nonsmokers. Pathogenic taxa such as *Treponema* and *Porphyromonas* correlated with increased cytokine levels and periodontal inflammation, positioning the EC microbiome as a distinct state with unique oral health risks, closely resembling that of conventional smokers [63].

Importantly, EC-induced oral dysbiosis disrupts microbial homeostasis, fostering pathogenic proliferation and metabolic alterations that contribute to a spectrum of oral pathologies, including periodontitis and oral carcinogenesis. For instance, EC aerosol exposure disrupted oral microbial balance by inhibiting the growth of commensal *Streptococcus* species (*S. sanguinis* and *S. gordonii*), while enhancing biofilm formation and attachment of the opportunistic pathogen *S. mutans*. *S. mutans* also showed increased hydrophobicity and coaggregation abilities, potentially facilitating its colonization and contributing to oral health issues. These results indicated that EC use might promote pathogenic bacteria while suppressing beneficial commensals, potentially leading to oral diseases like periodontitis and dental caries [64]. Similarly, EC use modified the oral microbiome in periodontitis patients, enriching *Filifactor*, *Treponema*, and *Fusobacterium*, with similarities to the microbial shifts seen in cigarette smokers. Increased abundance of *Porphyromonas gingivalis* and *Fusobacterium nucleatum* was observed, alongside elevated pro-inflammatory cytokines, including IFN-γ and TNF-α, which correlated with genera such as *Dialister* and *Selenomonas*. These changes exacerbated oral microbiome dysbiosis and periodontal disease progression [65]. Likewise, EC use increased oral microbiome dysbiosis, with higher α-diversity and altered β-diversity compared to non-users. Significant changes in microbial taxa, such as *Actinomyces*, *Rothia*, *Neisseria*, and *Enterococcus*, in subgingival sites mediated the association between EC use and gingival inflammation. These microbial shifts, along with changes in 71 KEGG pathways, highlighted the potential for EC use to contribute to periodontal disease through its impact on the oral microbiome [66]. Remarkably, EC exposure enhanced *S. aureus* attachment and biofilm formation on oral epithelial cells, promoting oral colonization. This was coupled with a reduction in immune response, as indicated by decreased IL-8, IL-6, and IL-1β secretion, and impaired clearance of *S. aureus*. EC exposure also increased COX2 expression, suggesting modulation of the oral inflammatory response. These effects potentially facilitated the progression of periodontitis and oral preneoplasia by promoting *S. aureus* colonization and altering immune function [67]. In addition, EC exposure disrupted oral microbiome diversity and metabolite profiles, with flavored EC aerosols containing nicotine leading to increased bacterial α-diversity. Metabolomics analysis revealed significant changes in metabolites, particularly those linked to oral cancer progression. These findings highlight the adverse impact of EC use on oral health, altering both microbiome composition and metabolic pathways in a 3D organotypic model of human oral mucosa [68].

In addition to the dysbiosis of oral microbiota induced by exclusive EC use, studies have further explored the synergistic effects of combined EC consumption with various types of tobacco products on oral microbial communities and their associations with oral pathologies. For instance, nicotine pouch and EC use, like conventional smoking, were associated with the presence of periodontopathogenic bacteria (*Porphyromonas gingivalis*, *Tannerella forsythia*, *Fusobacterium nucleatum*) in saliva, while no such pathogens were detected in non-tobacco users. Taken together, the findings implied that nicotine pouches and ECs might alter the oral microbiome and contribute to periodontal disease risk, though further quantitative studies are needed to confirm these preliminary results [69]. Additionally, EC altered the oral microbiome, with EC users showing increased *Veillonella* abundance and distinct β-diversity compared to non-EC users. Dual use of ECs and conventional cigarettes further enhanced microbial α-diversity and was associated with pathogenic taxa, underscoring the microbiome’s sensitivity to combined smoking behaviors [70]. Interestingly, both traditional CS and EC use modified the oral microbiome, though with distinct effects. Traditional cigarette smokers showed increased abundance of *Actinomyces* and *Prevotella*, while EC users exhibited higher *Veillonella* and lower *Porphyromonas* and *Peptostreptococcus* levels. Smoking disrupted the balance of oral flora, promoting anaerobic bacteria associated with dental decay and bad breath. ECs, while similarly impacting the oral microbiome, had a different effect compared to traditional cigarettes, necessitating further investigation into their links to oral diseases [71].

### Smokeless tobacco

Smokeless tobacco—composed of fermented plant materials, alkaline additives (e.g., sodium carbonate), and carcinogenic nitrosamines—differs fundamentally from combustible cigarettes and aerosolized ECs by delivering nicotine and toxins through direct mucosal contact rather than inhalation, a distinction that reshapes its biological interactions and uniquely perturbs oral microbial ecosystems through pH modulation, xenobiotic exposure, and epithelial barrier disruption. For instance, smokeless tobacco use induced oral mycobiome dysbiosis, with reduced diversity in users with oral lesions. The fungal genus *Pichia*, enriched in these users, correlated positively with *Starmerella* and *Fusarium* and negatively with *Alternaria*. Functional analysis revealed increased pathotrophic and saprotrophic fungal activities, suggesting disrupted fungal growth regulation. These alterations might contribute to oral carcinogenesis in smokeless tobacco users [72]. Similarly, smokeless tobacco aqueous extracts (STAEs) from various brands modulated the growth and viability of oral bacteria in a concentration-dependent manner, with some strains exhibiting inhibited growth and others enhanced growth. Snuff STAEs showed more toxicity to oral bacteria than snus. While tobacco-specific N-nitrosamines had minimal effects on bacterial growth and viability, STAEs disrupted the oral microbial balance by promoting certain bacterial strains and inhibiting others. These alterations in oral bacterial ecology might have implications for oral health [73]. Moreover, smokeless tobacco use significantly disrupted oral microbiota composition, with varying effects depending on dosage. Exposure to 250 mg of Grizzly snuff increased the abundance of *Firmicutes*, *Streptococcus*, *Actinomyces*, and other genera, while decreasing Bacteroidetes and Fusobacteria. Bacterial diversity was reduced at lower doses (2.5 mg) but increased at higher doses (250 mg). These findings highlight the impact of smokeless tobacco on oral microbial communities, indicating potential implications for oral health and disease development [74].

Smokeless tobacco use increased oral pathogenic bacterial diversity and promoted dysbiosis, particularly in users with oral premalignant lesions (OPL). Genera such as *Prevotella*, *Fusobacterium*, and *Veillonella* were enriched, alongside functional shifts in nitrogen, nucleotide, and energy metabolism pathways. The presence of HPV-16 and EBV further associated with OPL development, highlighting a cancer-promoting microbial profile in smokeless tobacco users [75]. In addition, smokeless tobacco altered the oral microbiome in OSCC patients, significantly enriching genera such as *Staphylococcus*, *Fusobacterium*, and *Campylobacter*, known for producing tobacco-specific nitrosamines. These microbial changes correlated with oncogenesis-related gene functions, highlighting the role of the oral bacteriome in oral carcinogenesis among smokeless tobacco users [76]. Similarly, smokeless tobacco consumption altered the oral microbiome, increasing inflammation-associated species and resembling the microbiome of OSCC patients. *Streptococcus* abundance distinguished healthy microbiomes from those of smokeless-tobacco users and OSCC sites. OSCC-associated microbiomes showed enrichment of Gram-negative genera such as *Prevotella*, *Capnocytophaga*, and *Fusobacterium*, linked to the lipopolysaccharide biosynthesis pathway, highlighting their potential as markers for oral cancer [77]. Moreover, Toombak, a traditional smokeless tobacco product widely consumed in Sudan, is composed of fermented tobacco leaves mixed with alkaline additives and was associated with elevated risks of OPLs and OSCC. Toombak use altered the oral microbiome, increasing *Staphylococcaceae*, *Corynebacterium_1*, and *Cardiobacterium* while reducing *Prevotella*, *Lactobacillus*, and *Candida*. *Corynebacterium_1* was enriched in early cancer stages and oral cancer samples, suggesting a role in carcinogenesis. Genera such as *Stenotrophomonas* and *Schlegelella* dominated the oral cancer microbiome of Toombak users, potentially contributing to metastasis and poor prognosis, highlighting microbiome modulations as a risk factor for oral cancer progression [78]. Likewise, a cross-sectional investigation examining Shammah consumption—a culturally entrenched smokeless tobacco practice in Arabian communities—demonstrated distinct taxonomic shifts in tongue dorsum microbial communities. Shammah used altered the tongue microbiome, with significant shifts in species composition, particularly enriching species with high acetaldehyde-production potential. Notably, *Rothia mucilaginosa*, *Streptococcus sp. oral taxon 66*, and *Actinomyces meyeri* were more abundant in shammah users, while *Oribacterium asaccharolyticum* was more abundant in non-users. These microbiome changes might contribute to oral carcinogenesis, highlighting the need for further investigation into the role of smokeless tobacco in altering oral microbial communities [79].

Several recent studies have conducted comparative analyses to investigate the differential impacts of smokeless tobacco products versus conventional CS on oral microbiota composition in human populations. Notably, evidence from a 2024 longitudinal cohort revealed that tobacco use altered the oral microbiome, with cigarette and smokeless tobacco users exhibiting increased bacterial diversity, including a higher abundance of *Firmicutes* and a lower abundance of Proteobacteria compared to non-users. Non-users had a greater relative abundance of *Actinomyces*, *Haemophilus*, *Neisseria*, *Rothia*, and *Veillonella*. Tobacco users also exhibited shifts in species abundance over time and the presence of opportunistic pathogens such as *Neisseria subflava* and *Porphyromonas endodontalis*, highlighting tobacco’s impact on oral microbial composition [80]. Likewise, 16S rRNA gene sequencing was employed in a cross-sectional analysis of the oral microbiome across three cohorts: traditional cigarette smokers, smokeless tobacco users, and healthy controls, aiming to characterize tobacco-associated microbial signatures. Smokers and smokeless tobacco users exhibited distinct oral microbiome profiles compared to healthy controls, with higher microbial diversity and significant compositional differences. Smokers showed increased abundance of *Fusobacterium spp*., *Saccharibacterium spp*., and S*huttleworthia*, while smokeless tobacco users had elevated levels of *Fusobacterium*, *Catonella*, and *Fretibacterium*. Functional pathways related to amino acid metabolism, including glutamate and aspartate biosynthesis, were enriched in both groups. These microbial and metabolic alterations highlighted the role of the oral microbiome in tobacco-related diseases, including oral cancer, and suggested its potential for diagnostic and therapeutic applications [81].

Collectively, these studies demonstrate that smokeless tobacco induces oral microbiome dysbiosis characterized by enrichment of pro-inflammatory taxa (e.g., *Fusobacterium* and *Prevotella*), depletion of symbiotic species (e.g., *Lactobacillus* and *Neisseria*), and activation of carcinogenesis-linked metabolic pathways (nitrogen metabolism and LPS biosynthesis), with dose-dependent effects and product-specific virulence. The convergence of acetaldehyde-producing fungi, oncoviral cofactors (HPV/EBV), and nitrosamine-enriched bacterial consortia establishes a polymicrobial carcinogenic milieu, necessitating functional metagenomic studies to disentangle microbial contributions to OSCC progression and targeted interventions to restore mucosal homeostasis.

### Reversibility of smoking’s impact on the oral microbiome

While smoking-induced dysregulation of the oral microbiome drives oral inflammation and carcinogenesis, the biological feasibility of post-cessation recovery—and whether this recovery can decouple between microbial communities and long-term health risks—remains a critical gap in guiding precision prevention strategies. Evidence indicated that CS reduced microbial α-diversity in the buccal mucosa but had limited effects on microbial diversity and composition in other oral and nasal sites. The oral microbiota exhibited marked site-specific heterogeneity, potentially contributing to its resilience against smoking-induced environmental perturbations [82]. Furthermore, tobacco smoking profoundly altered oral microbial composition, enriching *Bifidobacterium*, *Lactobacillus*, and the phylum *Actinobacteria* while depleting Proteobacteria, with notable taxa-level changes. These effects were consistent across African-American and European-American groups and were not observed in former smokers, highlighting the reversible nature of smoking-induced microbiota disruptions following cessation [83]. In addition, CS altered the tongue microbiome, decreasing *Neisseria* and *Capnocytophaga* while increasing *Streptococcus* and *Megasphaera*. These changes were associated with altered metagenomic pathways, including nitrate reduction and the tricarboxylic acid cycle. Former smokers’ microbiomes resembled those of never-smokers, suggesting reversibility of smoking-induced dysbiosis [84]. Notably, CS significantly altered the salivary microbiota, increasing the abundance of genera such as *Streptococcus*, *Prevotella*, and *Veillonella*, while decreasing *Neisseria*. These microbial shifts suggested that the salivary microbiome might be restored after smoking cessation. Linear Discriminant Analysis Effect Size revealed a microbial signature that could classify smokers and nonsmokers based on genus abundance. Further proteomics and metabolomics studies are necessary to explore bacterial endotoxins, xenobiotic metabolism, and their impact on bacterial interactions in the salivary microbiome [85]. Similarly, cigarette smoke exposure significantly altered the oropharyngeal microbiota composition, reducing its diversity, with over 60 taxa diminished after 6 months of exposure. This dysbiosis was reversible 3 months after smoke cessation. Lung infection with *Streptococcus pneumoniae* exacerbated lung damage and prolonged microbiota alterations compared to control groups. The data implied that while smoke exposure induced microbiota disruption and emphysema, structural lung damage alone did not maintain the altered microbiota, indicating that microbial shifts might not directly contribute to emphysema progression [86].

Several studies specifically examined the biological feasibility of EC discontinuation. For instance, EC use was shown to induce significant changes in the oral microbiome, with increased α-diversity in saliva and shifts in β-diversity in buccal mucosa compared to nonsmokers. EC users exhibited higher levels of *Veillonella* and *Haemophilus* in saliva, and a trend toward increased *Staphylococcus aureus* colonization in nasal samples. These changes were partially reversible with reduced vaping. The findings highlighted vaping’s impact on oral microbial diversity and composition, suggesting potential implications for oral health [87]. Furthermore, EC and CS significantly altered oral microbiome diversity and composition, increasing *Prevotellaceae* and decreasing *Neisseria* compared to nonsmokers. Smoking groups shared dominant phyla, including Proteobacteria, *Firmicutes*, and Bacteroidetes, but nonsmokers exhibited higher *Actinobacteria* and *Corynebacterium*, linked to distinct functional profiles. Smoking cessation restored the oral microbiome towards nonsmoker profiles, highlighting reversible effects on microbial structure and function [88].

## Dynamic interactions of cigarette smoke with the respiratory tract microbiota

Following its initial interaction within the oral cavity, inhaled cigarette smoke continues to exert profound effects along the respiratory tract, where it encounters another complex microbial ecosystem. The respiratory microbiota, extending from the nasopharynx to the alveoli, plays a central role in maintaining mucosal immunity and regulating inflammatory tone. Disruption of this delicate microbial balance by cigarette smoke leads to persistent immune activation, epithelial injury, and heightened susceptibility to infection and chronic airway disease. Understanding how smoking remodels the respiratory microbiome provides crucial insight into the mechanisms linking tobacco exposure to disorders such as COPD, asthma, and lung cancer.

**Fig. 3:**
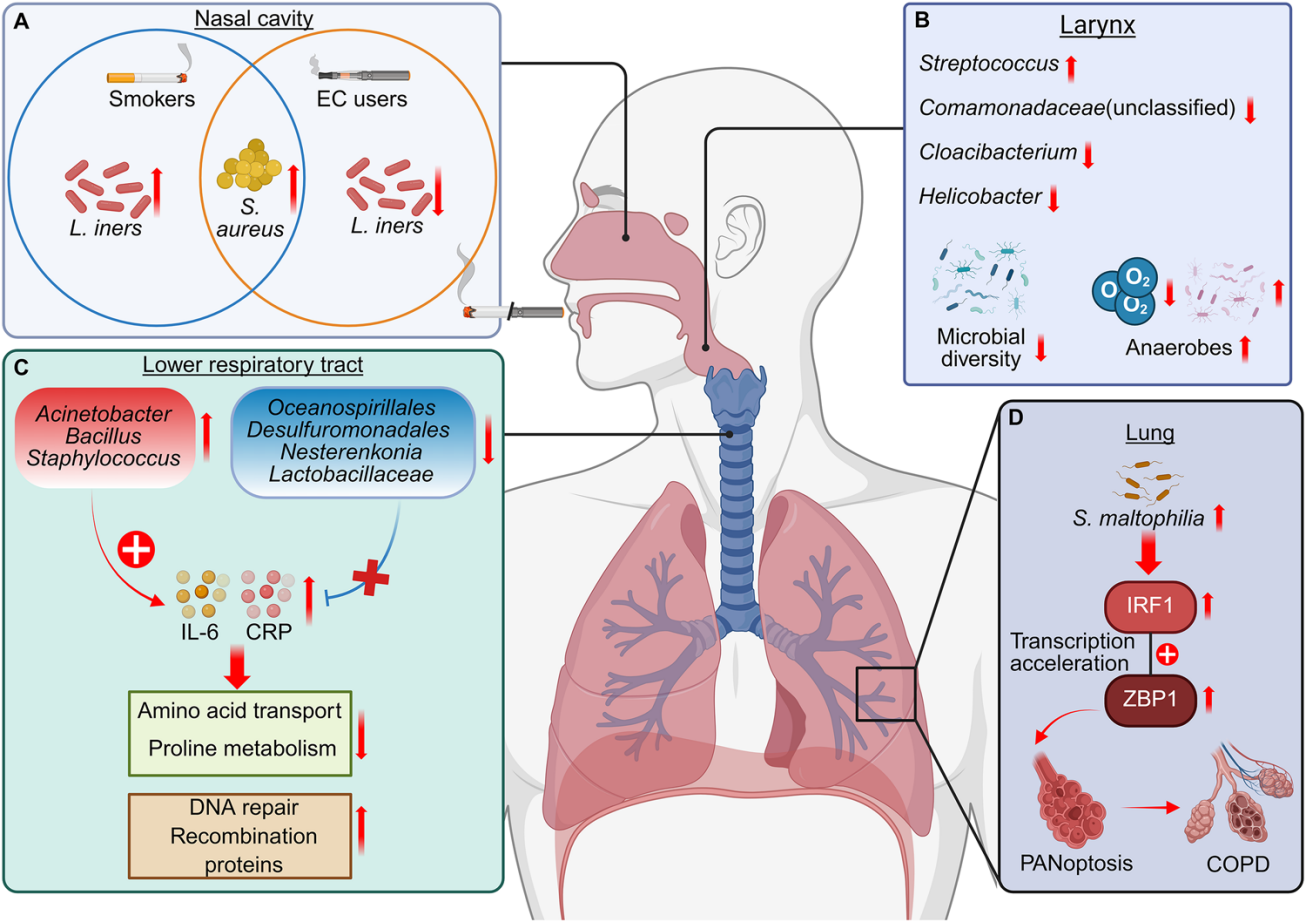
Cigarette smoke-induced microbial dysbiosis along the respiratory tract and its immunopathological consequences. **A** In the nasal cavity, both cigarette smoke (CS) and e-cigarette (EC) exposure increased the abundance of *Staphylococcus aureus*, while *Lactobacillus iners* was enriched in CS users but depleted in EC users. **B** In the larynx, smoking reduced microbial diversity, marked by an increase in *Streptococcus* and a decrease in anaerobic or microaerophilic taxa such as *Comamonadaceae* (unclassified), *Cloacibacterium*, and *Helicobacter*. **C** In the lower respiratory tract, CS promoted the overgrowth of pathogenic taxa (*Acinetobacter*, *Bacillus*, *Staphylococcus*) and suppressed beneficial commensals, including *Lactobacillaceae* and *Oceanospirillales*. These changes disrupted microbial metabolic functions—such as amino acid transport, proline metabolism, and DNA repair—and were associated with elevated inflammatory mediators IL-6 and C-reactive protein (CRP). **D** In the lungs, Stenotrophomonas maltophilia overgrowth activated the interferon regulatory factor 1 (IRF1) and Z-DNA-binding protein 1 (ZBP1) signaling cascade, triggering PANoptosis and worsening alveolar damage characteristic of chronic obstructive pulmonary disease (COPD). Collectively, these findings illustrate how smoking-induced respiratory dysbiosis drives chronic inflammation, impairs tissue repair, and accelerates disease progression. Created in https://BioRender.com.

The respiratory tract was long considered a sterile environment due to methodological constraints of traditional culture-based techniques. This paradigm shifted with the advent of high-throughput sequencing technologies, particularly 16S rRNA gene profiling and shotgun metagenomics, which unveiled a complex ecosystem harboring several hundred to over a thousand microbial species across the nasopharynx to alveoli [89]. Crucially, these culture-independent approaches enabled detection of fastidious anaerobes like *Prevotella* and *Fusobacterium*, whose dysregulation is now implicated in smoking-related pathologies. Leveraging these technological advances, recent multi-omics investigations have delineated smoking-induced remodeling of respiratory microbiota, with these dysbiotic changes further correlating to heightened risks of diverse respiratory pathologies (Table 2). For instance, CS significantly altered the lower respiratory tract microbiome, increasing the abundance of pathogenic bacteria such as *Acinetobacter*, *Bacillus*, and *Staphylococcus*, while decreasing beneficial taxa like *Lactobacillaceae*. Dysbiosis of Proteobacteria and *Firmicutes phyla* correlated with increased inflammation markers like IL-6 and C-reactive protein (CRP). Functional predictions showed disrupted microbial pathways, including amino acid transport and DNA repair, in smokers. This imbalance in the lung microbiome may disrupt immune homeostasis and contribute to inflammation, suggesting potential therapeutic targets such as probiotics (Fig. 3C) [16]. Moreover, CS disrupted microbiota, enriching *Streptococcaceae* in the oropharynx, with efficient transfer to germ-free mice. Upon influenza A virus infection, mice with cigarette smoke-associated microbiota exhibited greater weight loss and disease severity compared to controls. Microbiota changes induced by CS exposure, independent of structural lung damage, exacerbated influenza outcomes, highlighting the role of microbial dysbiosis in respiratory disease progression [90].

**Table 2:** Tobacco exposure reprograms the respiratory microbiota and triggers detrimental host responses.

Exposure/intervention type	Increased microbiota	Decreased microbiota	Functional/pathogenic alterations	Potential health implications	Ref
**Cigarette smoke**	*Acinetobacter, Bacillus, Staphylococcus*	*Lactobacillaceae*	Exacerbated inflammation, disrupted microbial pathways	Promotion of airway inflammation and infection susceptibility	[16]
**Cigarette smoke**	*Streptococcaceae*	-	Increased weight loss, worsened disease outcome upon influenza A virus infection	Exacerbation of pulmonary inflammation and immune dysregulation	[90]
**Cigarette smoke**	*S. maltophilia*	-	Induction of PANoptosis, suppression of organoid formation	Promotion of lung tissue damage and COPD progression	[91]
**Cigarette smoke**	Common oral commensals	-	Dysbiosis in lower airways; promotion of inflammatory and tumorigenic signaling pathways	Promotion of lower airway inflammation and susceptibility to infection	[15]
**Cigarette smoke**	*Streptococcus*	*Comamonadaceae* (unclassified) *, Cloacibacterium, Helicobacter*	Promotion of airway inflammation, reduced microbial diversity	Impaired mucosal resilience and altered laryngeal health	[92]
**Cigarette smoke**	Streptococcaceae, Spirochaetaceae	-	Altered lung microbial community in smokers with asthma	Exacerbation of asthma symptoms	[93]
**Cigarette smoke**	Streptococcus, Fusobacterium, *Prevotella, Haemophilus, Treponema, Enterobacteriaceae*	-	Dysbiosis linked to ARDS onset	Increased risk and severity of ARDS	[94]
**Electronic cigarette and cigarette**	*Staphylococcus aureus* (EC users and smokers)	*Lactobacillus iners* (EC users)	Dysbiosis of nasal microbiome, disrupted host–microbiota immune interactions	Impaired nasal mucosal immunity, increased susceptibility to respiratory pathogens	[95]
**Electronic cigarette and cigarette**	-	*Neisseria elongata, Haemophilus parainfluenzae* (smokers)	Smoking-associated reduction of bacterial species in lungs and oral cavity	Increased microbial vulnerability, increased risk of lung cancer and other disease risk	[96]
**Carbon nanotube (CNT) and cigarette smoke extract (CSE)**	Oral: *Acinetobacter* (CNT), *Staphylococcus, Aggregatibacter, Allobaculum, Streptococcus* (CSE), *Alkalibacterium* (CNT + CSE) Lung: *Firmicutes, Tenericutes* (CNT), Bacteroidetes (CSE)	Oral:- Lung: Proteobacteria, Bacteroidetes (CNT), Proteobacteria (CSE)	Promotion of oro-respiratory dysbiosis, induction of pro-inflammatory microbial shifts	Disruption of lung mucosal homeostasis, heightened airway inflammation	[97]

Substantial evidence has shown that CS-induced dysbiosis of the respiratory microbiota may drive COPD progression through disrupted microbial-host crosstalk and sustained airway inflammation. Notably, CS disrupted lung microbiota, promoting the expansion of *Stenotrophomonas maltophilia* in smoking-related COPD. *S. maltophilia* induced PANoptosis in alveolar epithelial cells via interferon regulatory factor 1 (IRF1)-mediated upregulation of Z-DNA Binding Protein 1 (ZBP1), impairing alveolar organoid formation. Targeting IRF1 mitigated *S. maltophilia*-induced lung injury, highlighting a link between microbial dysbiosis and emphysema progression in COPD (Fig. 3D) [91]. Furthermore, tobacco smoke induced lower airway dysbiosis in COPD, enriching oral commensals and promoting inflammatory pathways involving IL-17, IL-6, ERK/MAPK, and PI3K. These microbial and transcriptomic changes exacerbated inflammatory injury in early COPD, as corroborated in murine models, highlighting the interplay between smoking, airway microbiota, and disease pathogenesis [15].

Emerging studies suggest that CS-induced disruption of respiratory microbial communities may contribute to the pathogenesis of other respiratory diseases, including laryngitis, asthma and acute respiratory distress syndrome (ARDS). For instance, tobacco consumption reduced microbial diversity in the laryngeal microbiota, with notable shifts in the relative abundances of *Streptococcus*, *Comamonadaceae*, *Cloacibacterium*, and *Helicobacter*. Smokers exhibited less diversity compared to nonsmokers, while reflux status did not significantly impact microbial composition. The core laryngeal microbiota was dominated by *Comamonadaceae*, and increased *Streptococcus* abundance in benign vocal fold disease suggested its potential role in disease development. These findings highlight the impact of smoking on laryngeal microbiome structure and its possible contribution to chronic laryngitis (Fig. 3B) [92]. In addition, tobacco smoking in asthma patients increased bacterial diversity in the lungs compared to healthy nonsmokers, suggesting smoking influenced lung microbiota composition. However, smoking cessation did not significantly alter microbial diversity, indicating that while smoking exacerbated asthma symptoms and microbiome dysbiosis, cessation might not immediately restore microbial balance in the lungs [93]. Moreover, CS altered lung microbiota composition, with smokers showing enrichment of pathogens such as Streptococcus, Fusobacterium, *Prevotella*, *Haemophilus*, and *Treponema*. ARDS development after severe trauma was associated with microbial community shifts, including increased *Enterobacteriaceae* and specific taxa enriched in smokers, such as *Prevotella* and *Fusobacterium*. These results suggest smoking-related microbiota changes contribute to ARDS risk following trauma [94].

Comparative analyses of tobacco products have revealed divergent impacts on respiratory microbiota composition, with different tobacco products inducing distinct microbial alterations that may differentially modulate respiratory disease risks. EC and cigarette use induced significant dysbiosis in the nasal microbiome, with notable sex-dependent differences. Both EC users and smokers exhibited higher *Staphylococcus aureus* abundance, while *Lactobacillus iners*, a protective species, was less abundant in EC users. Dysbiosis also correlated with serum cotinine levels, indicating exposure to tobacco toxins. These results highlighted disrupted nasal immune responses and underscored the need for further research on the mechanisms by which ECs and smoking alter nasal immune homeostasis and microbiome composition, particularly considering sex as an important factor (Fig. 3A) [95]. Importantly, CS reduced bacterial abundance in the lung microbiome, with species such as *Neisseria elongata* and *Haemophilus parainfluenzae* significantly decreased. In contrast, EC use did not alter lung microbiota compared to never-smokers. Limited overlap between oral and lung microbiomes suggested the oral microbiome was not a reliable surrogate for smoking-related lung microbiome effects. These results underscored the distinct impact of cigarette smoke on microbial communities, potentially influencing disease risk [96]. Interestingly, a study investigating sub-chronic exposure to carbon nanotube (CNT) particles, cigarette smoke extract (CSE), and their combination on lung microbiota found that CSE altered lung microbiota, shifting from Proteobacteria to Bacteroidetes, while co-exposure with CNT resulted in mixed microbial effects, including increased Bacteroidetes and *Tenericutes*. CSE exposure also enriched pro-inflammatory oral genera such as *Streptococcus* and *Aggregatibacter*. These microbial changes correlated with disrupted lung mucosal homeostasis, highlighting the microbiome’s role in mediating smoking-induced respiratory toxicity [97].

## Smoking-driven restructuring of gastrointestinal microbial communities

Beyond the respiratory tract, cigarette smoke exerts profound systemic effects that extend to the gastrointestinal ecosystem—the largest and most metabolically active microbial reservoir in the human body. The gut microbiota not only governs nutrient metabolism and immune homeostasis but also serves as a critical interface linking environmental exposures to host physiology. Continuous ingestion and inhalation of tobacco-derived toxins perturb this finely tuned ecosystem, leading to widespread alterations in microbial composition, metabolite profiles, and mucosal barrier integrity. These changes reverberate through interconnected axes such as the gut–lung and gut–liver pathways, amplifying inflammatory and metabolic dysfunctions. Understanding how cigarette smoke remodels gut microbial networks is therefore essential to delineate its contribution to systemic diseases and to identify novel microbiome-targeted strategies for prevention and therapy.

The human gastrointestinal tract harbors diverse microbial communities that maintain a delicate equilibrium through intricate cross-feeding networks and host–microbe metabolic interactions. These microbial communities, comprising over 1000 bacterial species alongside archaea, fungi, and viruses, collectively orchestrate essential physiological functions ranging from nutrient metabolism to immune modulation [98, 99]. Building on previous discoveries, recent research has revealed that exogenous factors—particularly cigarette smoke exposure—can fundamentally remodel this dynamically balanced architecture via nicotine-mediated epigenetic pathways (Table 3). For instance, tobacco smoking altered gut microbiota composition, affecting taxa such as *Intestinimonas*, *Catenibacterium*, and *Ruminococcaceae*. Smoking also influenced the abundance of specific gut microbes, creating a positive feedback loop involving *Actinobacteria*, which might link parental smoking to early smoking initiation in children. Additionally, neurotransmitter-associated metabolites like tryptophan and tyrosine likely mediated the gut microbiome’s influence on smoking behavior. These results illuminated the bidirectional relationship between smoking and gut dysbiosis, underscoring the impact of tobacco use on microbial balance and its potential role in smoking behaviors [100]. Moreover, CS altered both host gene expression and the gut microbiome, with 71 differential species and 324 differentially expressed genes identified between smokers and nonsmokers. Smoking influenced the gut microbiome through changes in heme metabolism, particularly affecting *Bacteroides finegoldii* and *Lachnospiraceae bacterium 9_1_43BFAA*. Bidirectional mediation analysis revealed that smoking modulated gut microbes via gene expression, with key metabolites like porphobilinogen and bilirubin linked to microbial changes. These findings provided new perspectives on the role of heme metabolism in mediating the effects of smoking on the gut microbiome [20].

**Table 3:** Tobacco exposure-driven alterations in gut microbiota and associated pathophysiological effects.

Exposure/intervention type	Increased microbiota	Decreased microbiota	Functional/pathogenic alterations	Potential health implications	Ref
**Cigarette smoke**	*Intestinimonas, Catenibacterium*	*Ruminococcaceae, Actinobacteria*	Tryptophan/tyrosine-mediated microbial reinforcement of smoking	Intergenerational transmission of smoking behavior, gut dysbiosis	[100]
**Cigarette smoke**	*B. finegoldii, L. bacterium 9_1_43BFAA*	*-*	Heme metabolism alteration, transcriptome-mediated microbiota shifts	Gut dysbiosis	[20]
**Thirdhand smoke (THS)**	*-*	*Bifidobacterium*	THS-linked alterations in neonatal gut microbial composition	Infant gut development impairment	[102]
**Cigarette smoke**	*Lachnospiraceae sp*.	*-*	Increased *Muc2*/3/4 expression, altered cytokine levels, mucus barrier disruption	IBD susceptibility via mucus and immune disruption	[17]
**Cigarette smoke condensate (CSC)**	*Allobaculum*	*Eisenbergiella*	Impaired bactericidal function, Paneth cell granule abnormalities	IBD	[103]
**Cigarette smoke**	*Akkermansia, Flavonifractor*	*Lactobacillus, Barnesiella*	Downregulated inflammation-related pathways, promoted mucosal restoration	Attenuated ulcerative colitis severity	[104]
**Cigarette smoke**	*E. lenta*	*P. distasonis, Lactobacillus spp*.	Impaired gut barrier function, activated oncogenic pathways	CRC	[105]
**Cigarette smoke**	*E. shigella*	*Lachnospiraceae, Ruminococcaceae*	Oncogenic signaling–mediated disease progression, inflammation-driven disease progression	Type II colorectal neoplasms	[106]
**Cigarette smoke**	*Actinobacteriota* (lung)	*Patescibacteria, Campilobacterota* (intestine)	Microbiota-metabolite disruption, decrease SCFAs	LUAD, COPD	[21]
**Cigarette smoke**	*-*	*Akkermansia, Escherichia-Shigella*	Oxidative stress and inflammation, mitochondrial damage	COPD	[108]
**Cigarette smoke**	Eubacteriaceae	Ruminococcaceae, Desulfovibrionaceae, Rikenellacease	Modulated cytokine and adhesion molecule profiles	COPD, Crohn’s disease	[109]
**Cigarette smoke**	Akkermansiaceae, Rikenellaceae, Desulfovibrionaceae, Bacteroidaceae	-	Decreased plasma SCFAs, high-fiber diet–induced SCFA restoration	SCFA-based COPD therapy	[110]
**Tobacco carcinogen exposure**	Firmicutes, *Helicobacte, Alistipes, Odoribacter*	Bacteroidetes, *Lactobacillus, Akkermansia, Ruminococcus*	Decreased SCFAs, tobacco carcinogen-induced microbial dysbiosis	LUAD	[111]
**Cigarette smoke**	-	*Alistipes*	Cecal microbial dysbiosis	Weight loss in chronic smokers	[112]
**Cigarette smoke**	*Desulfovibrio, Bilophila*	*Clostridium, Turicibacter*	Altered amino acid/lipid metabolism, reduced insulin and leptin levels	Hyperglycemia, hypoinsulinemia, hypoleptinemia	[113]
**Cigarette smoke**	*Proteobacteria, Cyanobacteria, Verrucomicrobia, Epsilonbacteraeota*	*Firmicutes, Actinobacteria, Patescibacteria*	Increased cholesterol accumulation, altered bile acid homeostasis	Smoking-related hepatic disorders	[114]
**Cigarette smoke**	*Salmonella*	*Ligilactobacillus*	Dysregulation of lipid metabolism	Cigarette smoke-induced liver injury	[115]
**Cigarette smoke**	*Paraprevotella clara*	*-*	Decreased trypsin levels	Type 2 diabetes	[116]
**Cigarette smoke**	*Prevotella*	*Phycisphaera, C. asparagiforme*	Functional alterations of gut microbiota	Smoking-related hypertension	[117]
**Cigarette smoke**	*Cyanobacteria*	*Actinobacteria*	Increased exhaled CO levels, microbial dysbiosis, epithelial barrier dysfunction	Cardiovascular diseases	[118]

Recent advances have shed light on how thirdhand smoke (THS) exposure disrupts infant gut microbiota development by altering microbial colonization patterns and metabolic cross-talk during critical early-life stages. Exposure to THS during postnatal development significantly altered gut microbiome composition in mice, with minimal effects observed during pubescent or adult exposure. Postnatal THS exposure increased degradation pathways related to glycolysis and pyruvate decarboxylation, while decreasing coenzyme A biosynthesis and pyrimidine deoxyribonucleoside salvage pathways. These findings suggested that the gut microbiome was particularly sensitive to early-life THS exposure, highlighting the long-term impact of environmental tobacco toxins on microbial diversity [101]. Likewise, THS exposure in neonatal ICU infants altered gut microbiome composition, with lower α-diversity observed in infants from smoking households or those with higher surface nicotine levels. Reduced *Bifidobacterium* abundance correlated with increased urine cotinine and household smoking, suggesting tobacco-related exposures negatively impacted infant gut microbiome development [102].

### Smoking-driven gut microbiota destabilization exacerbates Crohn’s pathogenesis and activates colorectal oncogenic cascades

**Fig. 4:**
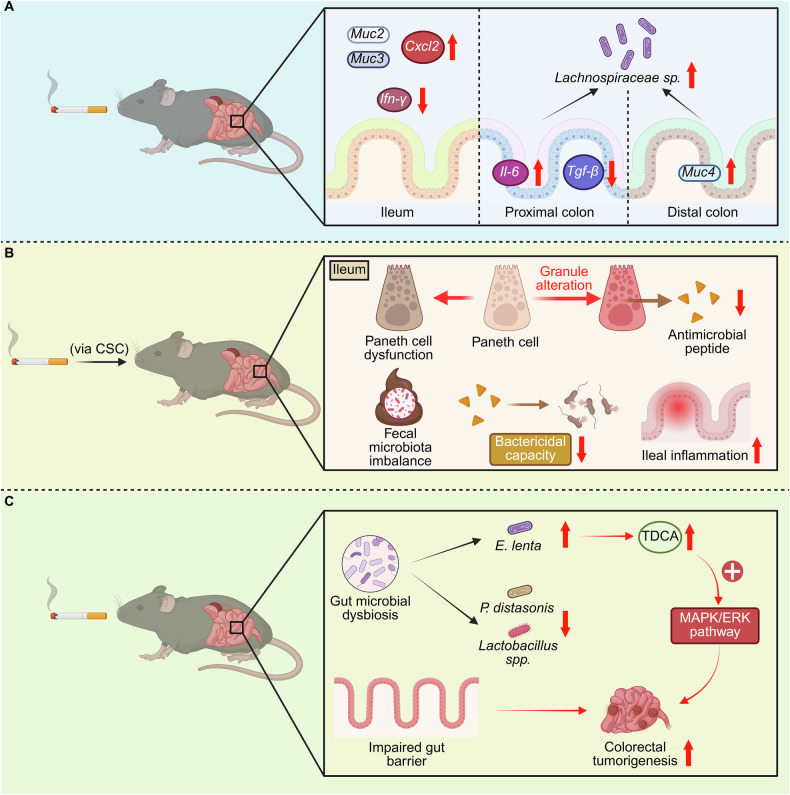
Smoking-induced gut microbial dysbiosis contributes to inflammatory bowel disease (IBD) and colorectal cancer (CRC). Cigarette smoke (CS) profoundly alters gut microbial composition and intestinal immune homeostasis, leading to chronic inflammation and tumorigenesis. **A** Chronic CS exposure increased the abundance of *Lachnospiraceae* species in the colon and upregulated mucus-associated genes (*Muc2*, *Muc3*, *Muc4*) and pro-inflammatory cytokines (*Cxcl2*, *Il-6*), while suppressing anti-inflammatory mediators such as interferon-gamma (*Ifn-γ*) and transforming growth factor-beta (*Tgf-β*). These changes disrupted mucosal barrier integrity and immune equilibrium across intestinal segments. **B** Exposure to cigarette smoke condensate (CSC) induced Paneth cell dysfunction in the ileum, resulting in granule abnormalities, reduced secretion of antimicrobial peptides, and decreased bactericidal capacity. This cascade led to fecal microbiota imbalance and promoted ileal inflammation, mimicking early pathogenic features of Crohn’s disease, a subtype of IBD. **C** CS-driven dysbiosis, characterized by an increase in *Eggerthella lenta* and depletion of *Parabacteroides distasonis* and *Lactobacillus* species, elevated taurodeoxycholic acid (TDCA) levels and activated the mitogen-activated protein kinase/extracellular signal-regulated kinase (MAPK/ERK) signaling pathway. This process compromised gut barrier integrity and promoted colorectal tumorigenesis, linking microbial alterations to carcinogenic signaling. Together, these findings illustrate how smoking-induced microbial and immune perturbations synergize to drive intestinal inflammation and malignant transformation. Created in https://BioRender.com.

Recent findings have highlighted that cigarette smoking disrupts gut microbial ecosystems, weakens intestinal barrier integrity, and triggers dysbiosis-driven inflammation. These alterations contribute to Crohn’s disease and activate carcinogenic pathways that promote colorectal tumorigenesis. Chronic smoke exposure markedly reshaped the gut microbiome, increasing *Lachnospiraceae sp*. activity in the colon and elevating *Muc2, Muc3*, and *Muc4* expression in the ileum and colon. It also altered immune mediators—upregulating *Cxcl2* and *Il-6* while suppressing *Ifn-γ* and *Tgf-β*—thereby disturbing mucosal homeostasis through concurrent shifts in microbiota, mucus composition, and cytokine signaling (Fig. 4A) [17]. Cigarette smoke condensate (CSC) aggravated inflammation and impaired Paneth cell function, reducing antimicrobial peptide secretion and bactericidal capacity. This led to fecal microbiota imbalance and increased susceptibility to bacterial injury. In IL-10(−/−) mice, CSC exposure induced severe enterocolitis, highlighting the role of smoke in disrupting intestinal equilibrium and promoting Crohn’s-like pathology (Fig. 4B) [103]. In experimental colitis, cigarette smoke modulated both the gut microbiome and colon transcriptome in a concentration-dependent manner. It altered dextran sodium sulfate–induced dysbiosis, affecting bacterial genera that could either resolve or sustain inflammation. Interestingly, epidemiological data indicated an inverse association between smoking and ulcerative colitis, suggesting complex tobacco–microbiota interactions that merit further study [104]. Beyond inflammation, smoke-induced dysbiosis facilitated colorectal cancer (CRC) progression. Tobacco exposure enriched *Eggerthella lenta* while depleting *Parabacteroides distasonis* and *Lactobacillus spp*., elevating bile acid metabolites such as taurodeoxycholic acid that activated MAPK/ERK signaling and promoted epithelial proliferation (Fig. 4C) [105]. Moreover, cigarette smoking increased the risk of type II colorectal neoplasms, with risk magnitude varying by gut enterotype. Smokers exhibited enrichment of carcinogenic *Escherichia–Shigella* and depletion of beneficial *Lachnospiraceae* and *Ruminococcaceae*. These shifts reinforced chronic inflammation and oncogenic signaling, linking smoke-related dysbiosis to colorectal tumorigenesis [106].

### Cigarette smoke disrupts gut–lung axis communication through microbial metabolite alterations driving pulmonary inflammation and cancer progression

**Fig. 5:**
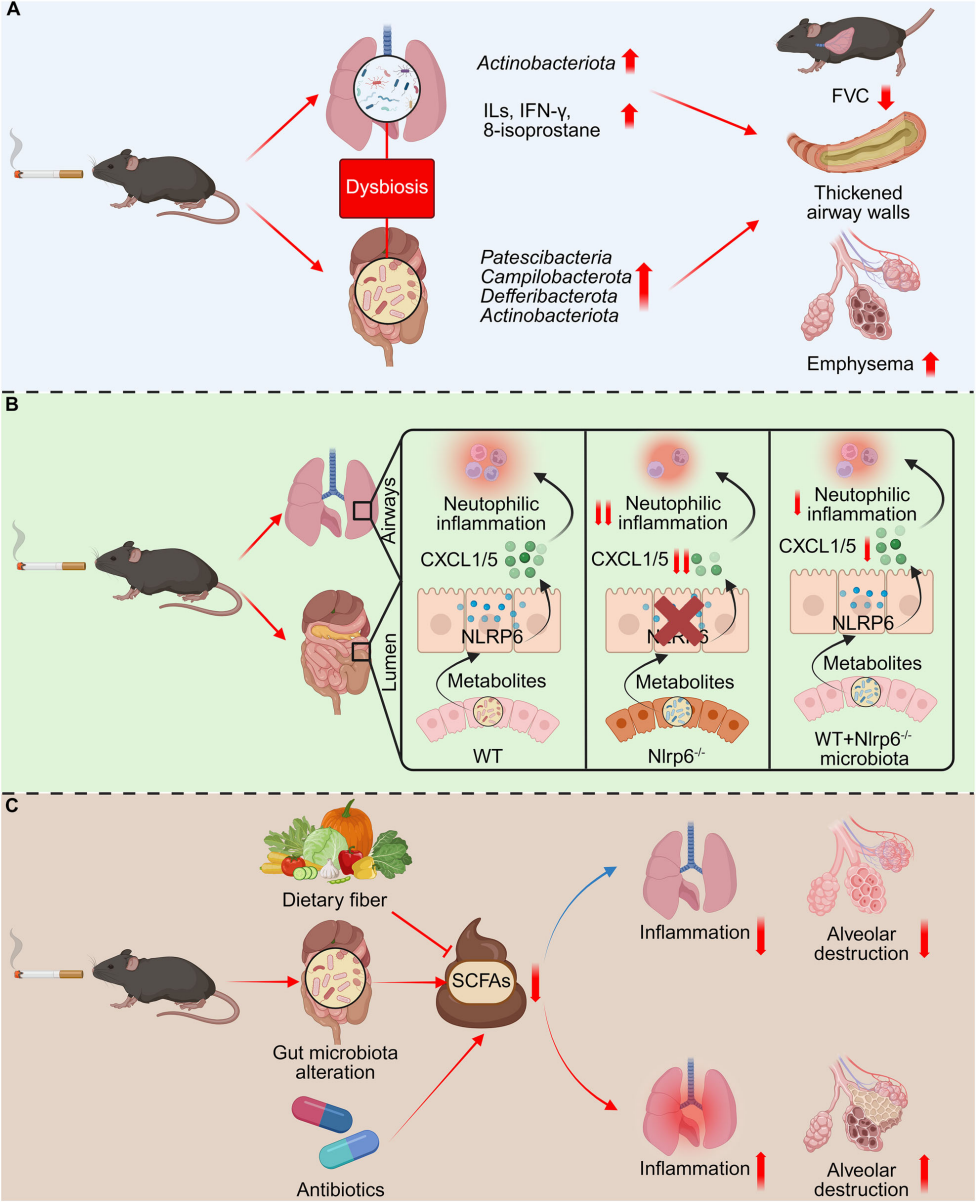
Cigarette smoke disrupts the gut–lung microbiome axis and promotes pulmonary inflammation. Cigarette smoke (CS) exerts bidirectional effects on the gut and lung microbiota, disrupting their homeostasis and amplifying inflammatory signaling along the gut–lung axis. **A** CS exposure induced microbial dysbiosis in both the respiratory and intestinal tracts. In the lungs, the relative abundance of *Actinobacteriota* increased, whereas in the gut, *Patescibacteria*, *Campilobacterota*, *Deferribacterota*, and *Actinobacteriota* decreased. These alterations were accompanied by elevated pro-inflammatory mediators, including interleukins (ILs), interferon-gamma (IFN-γ), and 8-isoprostane, as well as physiological impairments such as reduced forced vital capacity (FVC), airway wall thickening, and emphysematous changes, paralleling microbial and pathological patterns observed in patients with chronic obstructive pulmonary disease (COPD). **B** The NLR family pyrin domain containing 6 (NLRP6) inflammasome served as a pivotal regulator of smoke-induced pulmonary inflammation. Nlrp6^−^/^−^ mice displayed attenuated neutrophilic inflammation and reduced expression of chemokines CXCL1 and CXCL5. Fecal microbiota transplantation from Nlrp6^−^/^−^ donors into wild-type (WT) recipients recapitulated this anti-inflammatory phenotype, underscoring the role of NLRP6-dependent gut microbiota in shaping airway inflammatory responses through the gut–lung axis. **C** CS decreased fecal short-chain fatty acid (SCFA) production by depleting SCFA-producing microbes. Replenishing SCFAs via dietary fiber supplementation mitigated inflammation and alveolar destruction in smoke-exposed mice, whereas antibiotic-induced microbiota depletion exacerbated pulmonary inflammation and tissue injury. These findings reveal that maintaining SCFA metabolism and gut microbial balance may represent a promising therapeutic strategy for ameliorating smoking-related COPD. Created in https://BioRender.com.

Interestingly, accumulating investigations demonstrate that cigarette smoke distorts gut–lung axis communication by altering microbial metabolite profiles, creating a pro-inflammatory milieu that fuels pulmonary deterioration in COPD and promotes oncogenic niches in lung adenocarcinoma (LUAD). For instance, cigarette smoke exposure induced dysbiosis in both lung and intestinal microbiomes, with notable changes in bacterial diversity. Lung microbiome analysis revealed an increase in *Actinobacteriota*, while intestinal microbiome exhibited decreases in *Patescibacteria*, *Campilobacterota*, and others. These microbiome alterations correlated with lung function decline, including reduced forced vital capacity (FVC) and increased inflammation markers. Dysbiosis patterns observed in smoke-exposed mice were similar to those in severe COPD patients, suggesting a systemic impact of smoking on microbial balance and lung health (Fig. 5A) [21]. In addition, CS exposure altered gut microbiota composition in both wild-type and Nlrp6-deficient mice, with NLRP6 playing a critical role in controlling lung inflammation. Nlrp6-deficient mice exhibited impaired airway inflammation and neutrophil recruitment, while antibiotic treatment reduced CS-induced lung inflammation. Gut microbiota transferred from Nlrp6-deficient to wild-type mice attenuated lung inflammation, highlighting an Nlrp6-dependent gut-to-lung axis influencing pulmonary inflammation in response to smoking (Fig. 5B) [107]. Moreover, CS-induced COPD disrupted gut microbiota, reducing *Akkermansia* and *Escherichia-Shigella*, and activated the STAT3/NCOA4 pathway, promoting oxidative stress, inflammation, and ferritinophagy. Polyphyllin B restored microbial balance, inhibited STAT3/NCOA4 signaling, and ameliorated lung injury by enhancing ferritin and LC3 expression and mitigating mitochondrial damage, highlighting its therapeutic potential in smoking-related lung disease [108]. Additionally, tobacco smoke reduced intestinal microbial diversity and altered microbiota composition in mice, correlating with transcriptomic changes in the lung and ileum. Increased expression of genes such as MMP12 and SPP1 in the lung resembled COPD-like alterations, while intestinal gene changes (CD79B, PAX5) paralleled those in Crohn’s disease. These findings highlighted the interconnected impact of cigarette smoke on lung and gut inflammation, microbiota, and gene expression, suggesting shared mechanisms underlying COPD and intestinal disorders [109]. Likewise, tobacco smoking reduced SCFA production, with lower plasma SCFA concentrations observed in smokers, correlating with impaired lung function. Smoking altered gut microbiota and decreased fecal SCFAs, which could be modulated by dietary fiber or antibiotics. While SCFA modulation mitigated inflammation and alveolar destruction in a COPD mouse model, it had no effect in the elastase-induced model, suggesting SCFAs might influence COPD pathogenesis through their anti-inflammatory properties, offering potential therapeutic targets (Fig. 5C) [110]. Notably, tobacco carcinogen exposure led to significant changes in the gut and lung microbiomes, particularly in species like *Odoribacter*, *Alistipes*, *Akkermansia*, and *Ruminococcus*, which correlated with LUAD development and immunotherapeutic response. These changes were linked to decreased SCFAs such as propionic and butyric acid. Additionally, loss of Lcn2 expression disrupted microbiome composition, highlighting the role of microbial dysbiosis in tobacco-associated LUAD development. These results underscored the importance of microbiome dynamics in cancer progression and therapeutic responses [111].

### Tobacco smoke disrupts gut microbiota homeostasis driving metabolic dysfunction and multi-organ pathologies through inflammatory-metabolic axis dysregulation

Tobacco smoke exposure disrupts gut microbial homeostasis, triggering metabolic dysregulation and systemic pathologies, including weight loss, cardiovascular disease, diabetes mellitus, and hepatic dysfunction through microbiota-derived inflammatory mediators and metabolite imbalances. For instance, chronic cigarette smoke exposure altered the cecal microbiome, reducing microbial diversity and decreasing *Alistipes* abundance, a genus positively associated with body weight. Sex-specific differences in microbial composition were observed, with ovariectomy shifting the female microbiome to resemble that of males. These results suggested a link between smoke-induced microbiome changes and weight loss in smokers [112]. In addition, CS induced region-specific shifts in digestive tract microbiota, reducing beneficial genera such as *Clostridium* and *Turicibacter* while increasing harmful genera like *Desulfovibrio* and *Bilophila*. Functional predictions suggested impaired amino acid, lipid, and propionate metabolism alongside activated antioxidant pathways. These microbial alterations, coupled with hyperglycemia and reduced insulin and leptin levels, highlighted the role of smoking-induced gut dysbiosis in chronic disease risk [113]. Furthermore, tobacco smoking disrupted gut microbiota composition and hepatic metabolism, increasing cholesterol accumulation and altering bile acid homeostasis, particularly under high-fat diets. Changes in primary bile acid distribution and reduced CYP8B1 expression might contribute to smoking-induced insulin resistance and metabolic dysfunction. These findings highlighted the microbiome’s role in mediating smoking-related hepatic disorders [114]. Likewise, tobacco smoke exposure induced gut microbiota imbalances and liver injury, with significant changes in lipid metabolism-related gene expression in the liver. *Salmonella* correlated with the upregulation of lipid metabolism genes, while *Ligilactobacillus* showed opposite trends. These results underscored the coordinated regulation of lipid metabolism by gut microbiota and liver function, revealing key gut–liver interactions affected by smoking [115]. Notably, CS has been causally linked to metabolic diseases, with gut microbiota acting as a key mediator. Mendelian randomization analysis revealed that smoking influenced gut microbiota composition, particularly *Paraprevotella clara*, which significantly mediated the association between smoking and type 2 diabetes. The data highlighted the genetic and microbial interplay in smoking-induced metabolic disorders, underscoring the gut microbiome’s critical role in disease development [116]. Moreover, smoking cigarettes exacerbated gut microbiota dysbiosis in hypertensive individuals, reducing microbial α-diversity and shifting enterotypes toward *Prevotella*-dominant profiles. Smokers with hypertension showed reduced enrichment of beneficial taxa such as *Phycisphaera* and *Clostridium asparagiforme*, alongside altered microbial functions, highlighting the detrimental impact of smoking on gut health and its potential role in cardiovascular risk [117]. Importantly, CS altered intestinal microbiota, with *Actinobacteria* negatively correlating with pack-years and *Cyanobacteria* positively correlating with CO levels. Smoking cessation increased Bacteroidetes, decreased *Firmicutes*, and modestly raised α-diversity, which was inversely associated with heart rate, systolic blood pressure, and CRP. These outcomes suggested links between smoking, microbiota composition, and cardiovascular risk factors [118].

### Diet-pharma-cessation-microbe axis mitigates tobacco-driven gut dysbiosis

**Fig. 6:**
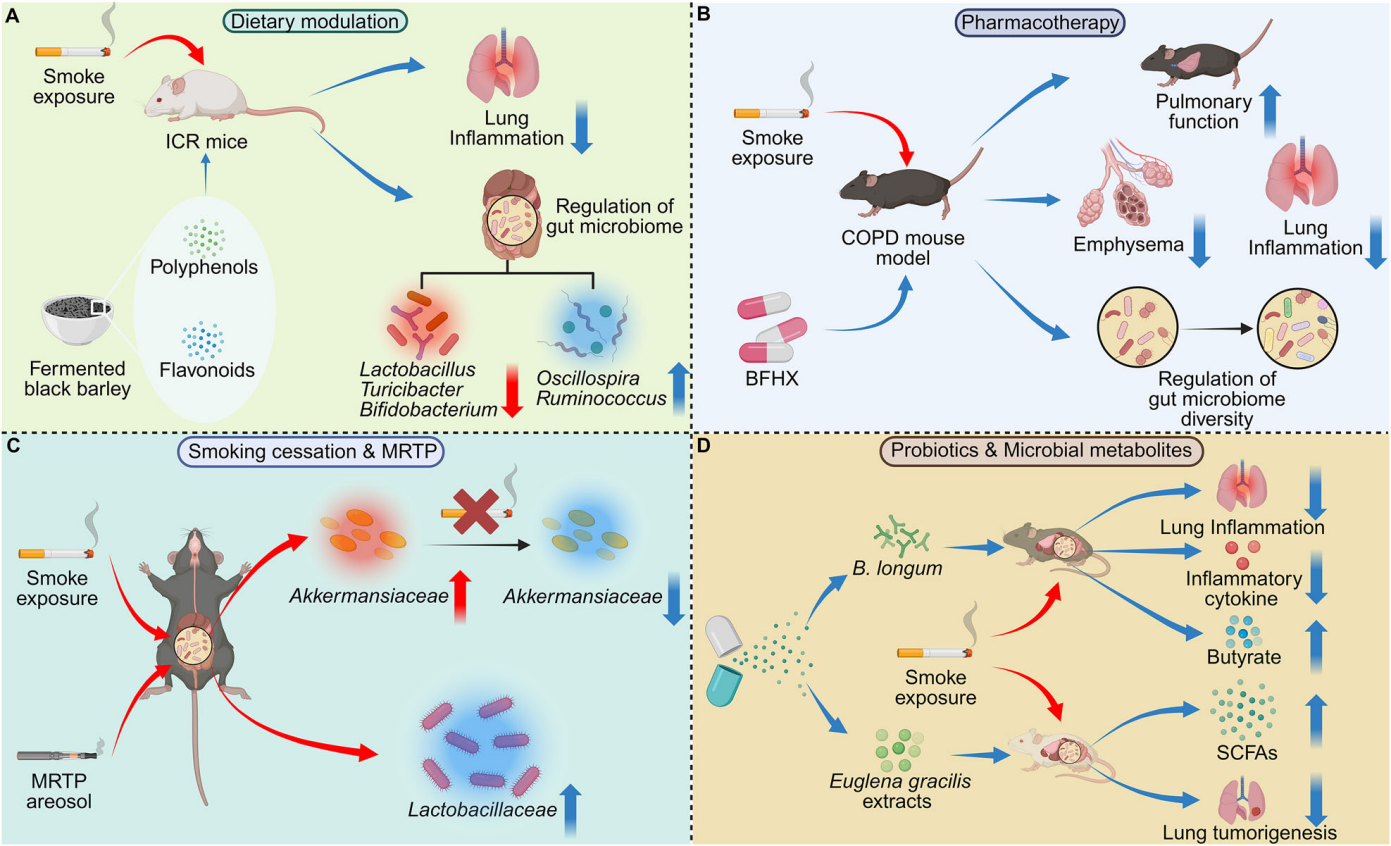
Dietary, pharmacological, cessation-based, and microbial interventions mitigate cigarette smoke-induced gut dysbiosis and pulmonary pathology. Cigarette smoke (CS) exposure perturbs the gut–lung axis, yet several interventional strategies have shown potential in restoring microbial balance and reducing inflammation. **A** Dietary supplementation with fermented black barley, rich in polyphenols and flavonoids, attenuated smoke-induced lung inflammation in ICR mice and helped re-establish gut microbial homeostasis. This intervention decreased the abundance of *Lactobacillus*, *Turicibacter*, and *Bifidobacterium* while enriching *Oscillospira* and *Ruminococcus*, suggesting a regulatory effect on the gut microbiome. **B** In a CS-exposed chronic obstructive pulmonary disease (COPD) mouse model, administration of Bufei Huoxue (BFHX) capsules improved pulmonary function, alleviated emphysema and lung inflammation, and restored gut microbial diversity, indicating that gut–lung axis modulation might underlie its therapeutic efficacy. **C** Smoking cessation reversed smoke-associated microbial alterations, notably reducing the relative abundance of *Akkermansiaceae*. Transition to modified-risk tobacco products (MRTPs), such as CHTP 1.2 aerosols, increased *Lactobacillaceae* levels, implying partial recovery of microbiota composition compared with conventional cigarette exposure. **D** Probiotic and microbial metabolite supplementation provided additional protective effects. Oral administration of *Bifidobacterium longum* suppressed lung inflammation, reduced inflammatory cytokine expression, and restored cecal butyrate levels. Similarly, *Euglena gracilis* extracts enhanced short-chain fatty acid (SCFA) production and inhibited tobacco carcinogen-induced lung tumorigenesis by promoting cell cycle arrest and apoptosis. Created in https://BioRender.com.

Multimodal interventions encompassing dietary modulation, pharmacotherapy, smoking abstinence, probiotic supplementation, and microbial metabolite administration demonstrate efficacy in counteracting tobacco-induced gut dysbiosis and restoring microbial equilibrium. For instance, fermented black barley, rich in polyphenols and flavonoids, mitigated these effects by restoring microbial diversity, decreasing *Lactobacillus*, *Turicibacter*, and *Bifidobacterium* abundances, and increasing *Oscillospira* and *Ruminococcus*. It also alleviated smoking-induced metabolic disturbances, highlighting its potential in counteracting gut and systemic disruptions associated with smoking (Fig. 6A) [119]. Additionally, cigarette smoke exposure in a COPD mouse model altered the intestinal microbiome, with changes in diversity, composition, and metabolism, including lysine degradation and phenylalanine metabolism. Bufei Huoxue capsule (BFHX) treatment improved pulmonary function and reduced inflammation, while also modulating gut microbiota. The treatment dynamically regulated microbiome diversity and composition, highlighting a potential therapeutic mechanism for COPD through gut–lung interactions (Fig. 6B) [120]. Notably, cigarette smoke exposure induced significant shifts in the intestinal microbiome, notably increasing *Akkermansiaceae* abundance, which was reversed upon cessation. Switching to modified-risk tobacco products (MRTPs) like carbon-heated tobacco product 1.2 (CHTP 1.2) increased *Lactobacillaceae* abundance. These microbial changes suggested that CS altered gut microbiome composition and gene expression, with potential implications for gut function and disease pathogenesis. These findings highlighted the role of the microbiome in mediating smoking-related health risks and the potential for MRTP to modulate these effects (Fig. 6C) [121]. Likewise, CS reduced bacterial diversity in the duodenal mucosa-associated microbiota, increasing *Streptococcus*, *Veillonella*, and *Rothia* abundance while decreasing *Prevotella* and *Neisseria*. These microbiota changes persisted partially in former smokers, suggesting incomplete restoration post-cessation and potentially contributing to smoking-related gastrointestinal diseases [18]. By contrast, tobacco consumption significantly altered gut microbiota composition, with current smokers showing increased Bacteroidetes and decreased *Firmicutes* and Proteobacteria compared to never-smokers. β-diversity analysis revealed significant differences between current and never-smokers, as well as between former and current smokers. No significant differences were found between never and former smokers, suggesting that smoking cessation did not immediately revert microbiota composition to baseline [122]. Interestingly, cigarette smoke exposure depleted beneficial gut microbiota, including *Bifidobacterium longum*, and impaired SCFA production, particularly butyrate. Administration of *B. longum*, regardless of acetate production capacity, alleviated cigarette smoke-induced lung inflammation, reduced inflammatory cytokine and adhesion factor expression, and restored cecal butyrate levels. This highlighted the potential of *B. longum* probiotics in mitigating cigarette smoke-induced gut–lung axis dysregulation and inflammation (Fig. 6D) [123]. Furthermore, *Euglena gracilis* extracts counteracted tobacco carcinogen-induced lung tumorigenesis by modulating gut microbiota and increasing metabolites such as triethanolamine, salicylate, and SCFAs like acetate, propionate, and butyrate. These metabolites induced cell cycle arrest and apoptosis in lung carcinoma cells, linking gut microbiota alterations to anti-cancer activity against tobacco smoke-related carcinogenesis [124].

## Challenges

Recent advances have illuminated the multifaceted ways in which cigarette smoke reconfigures microbial composition, functional capacity, and metabolic activity across body sites. Yet, the translation of these associations into mechanistic understanding remains hindered by methodological variability, inter-individual heterogeneity, and the intrinsic complexity of host–microbe–environment interactions. A significant challenge in studying the impact of smoking on the microbiome is the inherent variability in microbiome composition between individuals. This variability arises from multiple factors, including genetics, diet, environment, and lifestyle. Genetic differences can shape immune responses and microbial populations, while diet influences microbial diversity by providing nutrients that favor certain bacteria. Environmental exposures, along with lifestyle behaviors such as exercise, alcohol consumption, and medication use, also significantly affect microbiome composition [125–127]. This natural heterogeneity complicates efforts to isolate the specific effects of smoking, as it becomes difficult to discern whether observed changes in microbial composition are due to smoking itself or interactions between smoking and other factors. For example, smokers with different baseline microbiome compositions may exhibit distinct responses to smoking. To overcome this challenge, future studies should incorporate larger, demographically diverse cohorts that better represent population-level diversity. Employing statistical frameworks capable of adjusting for microbial covariates, such as host genetics, diet, and environmental factors, will enhance the robustness of findings. Additionally, integrating host genotyping and dietary profiling with microbiome data will refine stratification strategies and facilitate the development of individualized microbial risk signatures linked to tobacco exposure. Multi-omics approaches, combining genomics, transcriptomics, and metabolomics, could also provide a comprehensive understanding of the interactions between smoking and microbial communities [128, 129].

Sampling microbial communities presents another significant challenge, as it directly impacts the accuracy and reliability of study results. In gut microbiome studies, fecal samples are commonly used as proxies for intestinal microbial populations. However, substantial differences exist between the fecal microbiota and mucosal microbiota across various regions of the intestine. This discrepancy limits the representativeness of fecal samples, especially when studying localized conditions or disease states. Microbial communities in different sections of the gut, such as the small intestine and colon, vary in species composition and functional capacity, meaning that fecal samples may not fully reflect the diversity and dynamics of the intestinal microbiome [130–132]. The respiratory microbiome presents additional challenges due to its low microbial biomass, which makes it more susceptible to contamination during both sampling and sequencing. The relatively small number of microorganisms in the respiratory tract, compared to other body niches, requires highly sensitive methods for detecting and characterizing microbial species. Contamination from external sources or the upper airway can also interfere with analysis, leading to inaccurate data interpretation [133, 134]. To address these challenges, future research must focus on methodological innovations. Standardizing minimally invasive mucosal biopsies, spatially resolved metagenomics, and contamination controls—such as synthetic spike-in standards and dual-indexed barcoding—will enhance the reproducibility and accuracy of microbiome data [135–137]. Additionally, noninvasive yet site-specific sampling strategies should be developed to improve ecological fidelity and ensure that microbial communities are adequately represented [138, 139].

While animal models provide invaluable insights into microbial colonization and host–microbe interactions, they exhibit notable limitations when translating findings to humans. Murine studies, in particular, often employ smoke exposure protocols that differ significantly from human smoking behaviors, such as variations in smoke composition, exposure methods, and involuntary exposure patterns. These differences complicate the ability to assess smoking-induced microbiome alterations and associated disease mechanisms in humans [140, 141]. To enhance the translational relevance of animal studies, future research should emphasize the use of humanized gnotobiotic models, which are colonized with microbiota derived from well-characterized cohorts of smokers and nonsmokers. Combined with controlled environmental exposure systems—such as whole-body smoke chambers—and longitudinal tracking of host physiological and immunological responses, these models could offer a more accurate representation of real-world smoking conditions [142, 143]. Additionally, integrating advanced imaging techniques with microbiome analyses could provide deeper insights into the spatial distribution and interactions of microbial populations within different anatomical niches.

Another area that requires further investigation is the potential role of microbial metabolites in disease progression. It has been increasingly recognized that microbial dysbiosis, driven by smoking, can alter the production of key metabolites that influence host health. Understanding how microbial metabolites contribute to disease onset and progression is crucial for developing therapeutic interventions. To explore this, future studies should incorporate advanced metabolomics techniques to profile microbial metabolites produced in response to smoking. Animal models colonized with microbiota derived from smokers could help elucidate the direct contributions of these metabolites to disease development. Additionally, integrating bacterial, viral, and fungal analyses with metabolite profiling could uncover critical components that influence disease pathophysiology. Identifying and functionally characterizing these microbial metabolites may lead to novel therapeutic strategies aimed at modulating the microbiome to mitigate disease risks and improve smoking cessation outcomes [144, 145].

Finally, the integration of microbiome-based interventions offers a promising avenue for combating the adverse effects of smoking on health. Strategies such as probiotics, prebiotics, and dietary modifications, which aim to restore microbial balance, could be explored as adjunct therapies to smoking cessation programs. Moreover, microbiome-targeted therapies, including the use of antimicrobial peptides or specific bacteriophages, may provide more direct means of modulating microbial communities to reduce disease risk. Clinical trials investigating these interventions are needed to assess their efficacy in preventing or treating smoking-related diseases, such as periodontitis, COPD, and cancer.

## Conclusions and perspectives

CS imposes widespread systemic consequences not only through its direct toxicological insults but also by reshaping microbial ecosystems that are fundamental to host homeostasis. Mounting evidence indicates that smoking-induced dysbiosis across the oral, respiratory, and gut microbiota perturbs immune–metabolic balance, thereby contributing to the onset and progression of inflammatory, metabolic, and neoplastic diseases. Although multi-omics technologies have delineated characteristic microbial signatures associated with tobacco exposure, key questions regarding causality, reversibility, and therapeutic applicability remain unresolved. Progress is hindered by the confounding effects of host genetics, diet, and environment, the heterogeneity of microbial sampling and sequencing methodologies, and the limited fidelity of rodent models in recapitulating human smoking behaviors. Future investigations should integrate longitudinal human cohorts with mechanistic animal studies colonized by smoker-derived microbiota, which may provide causal insight into how specific taxa or metabolites mediate disease trajectories. Moreover, dynamic multi-omics profiling that couples microbial composition with host immune and metabolic states will be pivotal to identifying keystone species and functional nodes amenable to therapeutic manipulation.

Beyond mechanistic understanding, delineating smoking-related microbial dysbiosis opens a new avenue for precision microbiome-based interventions. Strategies such as targeted probiotic supplementation, prebiotic and dietary modulation, engineered bacteriotherapies, or postbiotic metabolite administration hold promise in restoring microbial equilibrium and attenuating inflammation in smokers. Yet, the translation of these insights into clinical application remains challenging. Inter-individual variability in microbiome composition, transient colonization following microbial supplementation, and the lack of standardized endpoints to evaluate therapeutic efficacy collectively impede progress. Furthermore, the causal hierarchies linking microbial shifts to host pathology are still incompletely defined, complicating the design of rational microbiota-targeted interventions. Overcoming these barriers will require harmonized analytical frameworks, integrative computational models, and ethically guided clinical trials that align microbiome modulation with personalized disease risk stratification.

As the field evolves, coupling microbial ecology with systems-level host responses may transform our understanding of tobacco-related pathogenesis. This integrative perspective has the potential to advance microbiota-centered prevention and treatment strategies, ultimately complementing traditional smoking-cessation and public health efforts to alleviate the global burden of tobacco-induced disease.

## Data Availability

No datasets were generated or analysed during the current study.
